# A hybrid demultiplexing strategy that improves performance and robustness of cell hashing

**DOI:** 10.1093/bib/bbae254

**Published:** 2024-06-03

**Authors:** Lei Li, Jiayi Sun, Yanbin Fu, Siriruk Changrob, Joshua J C McGrath, Patrick C Wilson

**Affiliations:** Gale and Ira Drukier Institute for Children’s Health, Weill Cornell Medicine, 413 E. 69th Street, New York, NY 10021, United States; Center for Applied Bioinformatics, St. Jude Children’s Research Hospital, 262 Danny Thomas Place, Memphis, TN 38105, United States; Gale and Ira Drukier Institute for Children’s Health, Weill Cornell Medicine, 413 E. 69th Street, New York, NY 10021, United States; Gale and Ira Drukier Institute for Children’s Health, Weill Cornell Medicine, 413 E. 69th Street, New York, NY 10021, United States; Gale and Ira Drukier Institute for Children’s Health, Weill Cornell Medicine, 413 E. 69th Street, New York, NY 10021, United States; Gale and Ira Drukier Institute for Children’s Health, Weill Cornell Medicine, 413 E. 69th Street, New York, NY 10021, United States; Gale and Ira Drukier Institute for Children’s Health, Weill Cornell Medicine, 413 E. 69th Street, New York, NY 10021, United States

**Keywords:** cell hashing, scRNA-seq, hybrid strategy, single cell demultiplexing, sample pooling, HTOreader

## Abstract

Cell hashing, a nucleotide barcode-based method that allows users to pool multiple samples and demultiplex in downstream analysis, has gained widespread popularity in single-cell sequencing due to its compatibility, simplicity, and cost-effectiveness. Despite these advantages, the performance of this method remains unsatisfactory under certain circumstances, especially in experiments that have imbalanced sample sizes or use many hashtag antibodies. Here, we introduce a hybrid demultiplexing strategy that increases accuracy and cell recovery in multi-sample single-cell experiments. This approach correlates the results of cell hashing and genetic variant clustering, enabling precise and efficient cell identity determination without additional experimental costs or efforts. In addition, we developed HTOreader, a demultiplexing tool for cell hashing that improves the accuracy of cut-off calling by avoiding the dominance of negative signals in experiments with many hashtags or imbalanced sample sizes. When compared to existing methods using real-world datasets, this hybrid approach and HTOreader consistently generate reliable results with increased accuracy and cell recovery.

## Introduction

Recent technical advances in single-cell sequencing have greatly benefited biological research by enhancing our ability to investigate cellular mechanisms of homeostasis and disease in a more precise, high-resolution, and multi-omic fashion [1–3]. In the past decade, numerous single-cell methods have been proposed to improve the quality, magnitude, modality, and economy of single-cell experimental approaches [1, 4–10]. Among them, sample pooling and demultiplexing can greatly reduce the per-cell cost and have therefore been extensively used.

Sample multiplexing approaches in single-cell sequencing were first proposed in 2015 [11]; since that time, many variants of this approach have been developed [12]. For single-cell RNA-seq, current demultiplexing methods fall into several major technical solutions, including nucleotide barcode-based methods (e.g. cell hashing, MULTI-seq, ConA-based sample-barcoding strategy, BART-Seq, and ClickTags), natural genetic variation-based methods (e.g. demuxlet, scSplit, Vireo, and Souporcell), and vector-based barcoding methods [8–10, 13–19]. Among them, cell hashing has been one of the most popular approaches due to its ease of compatibility with existing experimental and data processing pipelines [20]. Specifically, hashing involves staining cells from each sample with uniquely barcoded antibodies [also called hashtags, or hashtag oligonucleotides (HTOs)] that recognize ubiquitously expressed surface antigens, such as CD298 and β2-microglobulin [21]. The HTO-labeled cells can then be pooled and prepared for single-cell sequencing, and the sample identities of each cell can be determined based on the relative enrichment level of each hashtag using computational approaches.

Despite the widespread usage of cell hashing, certain issues exist due to the nature of this technique. Ideally, cells from a hashing experiment should be divided into two groups: negatives (no signal to any hashtag) and singlet positives (positive signal to one hashtag). However, interpreting the results of real-world datasets can be more complex and present additional challenges. First, a background signal may be present for a given hashtag, producing false singlets (unlabeled cells categorized into a random singlet). Furthermore, cross-contamination caused by hashtag spill-over during sample pooling often leads to the presence of false doublets (single cells positive for multiple hashtags), whose frequency increases with the number of hashtags being used. Finally, improper staining conditions can occasionally cause hashtag inefficiency, resulting in a significant number of negative cells (cells with no hashtag signal). These problems are common among real-world datasets and difficult to avoid experimentally.

To demultiplex cell hashing experiments, numerous computational methods have been developed. Among them, HTOdemux (Seurat), Multi-seq, GMM_Demux, BFF, and DropletUtils have been shown to work well with benchmark datasets and therefore gained widespread usage [13, 22–25]. Among these methods, HTOdemux groups cells based on their cell hashing profiles using k-medoids clustering, while MULTI_seq employs a similar heuristic approach that defines positives and negatives based on local maxima of the probability density of central-log-transformed cell hashing counts. DropletUtils assumes a specified difference (log-fold change) between negative and positive cell hashing profiles, GMM-Demux decomposes centered log-ratio (CLR)-transformed HTO counts into two Gaussian distributions representing positive and negative groups and BFF, the most recent method, classifies cells in the Bimodal Quantile Normalization (BQN) space developed alongside BFF models. Despite using different mathematical models, these methods all function by determining the appropriate cutoff for each hashtag based on the bimodal distribution of hashtag counts (or normalized counts) [24]. As a result, these methods can perform poorly in real-world datasets when actual distributions differ significantly from the assumptions made by the models. For example, HTOdemux, GMM-Demux, Multi-seq, and DropletUtils have been reported to perform poorly on datasets that have insufficient distinction between two peaks (even multiple peaks) of hashtag distributions [23, 24]. Together, the performance of current demultiplexing methods for cell hashing can vary significantly among different real-world datasets.

Recently, multiple genetic variation-based demultiplexing methods have been developed that determine donor identity by analyzing natural single nucleotide polymorphisms (SNPs) within sequenced DNA or RNA. Here, we propose a hybrid strategy that improves the overall performance of existing approaches by integrating the results of SNP-based demultiplexing with cell hashing. This approach is further complemented by HTOreader, a novel tool that improves the cutoff calling of individual hashtag distributions in real-world datasets that have imbalanced sample sizes or use many hashtags. For both benchmark and real-world datasets, our hybrid strategy significantly increases accuracy and cell recovery rate when compared to existing demultiplexing approaches for cell hashing alone. Significant benefits are also observed in datasets that have a low recovery rate due to poor hashtag staining quality. In applying our hybrid approach to two datasets from the same group of human donors, one stained with hashtag and one without, we find that performance remains consistent even among unstained cells; this indicates that reagent costs can be substantially decreased by only hashing a small number of cells from each sample. Furthermore, by integrating results from two methods based on completely different mechanisms (hashing and SNPs), this hybrid approach is inherently self-validating; potential errors that arise from one method can be easily identified by the other, making hybrid demultiplexing more reliable than other unimodal methods. Finally, rather than proof of concept, we demonstrate the workflow and performance of this strategy in a real-world B cell study. Together, our hybrid strategy provides a robust, accurate, and cost-effective solution for demultiplexing single-cell RNA sequencing datasets.

## Materials and methods

### Reagents

**Table:** 

Reagent	Source	Identifier
TotalSeq™-C0154 anti-human CD27 Antibody	BioLegend	Cat# 302853
TotalSeq™-C0187 anti-human CD79b (Igβ) Antibody	BioLegend	Cat# 341417
TotalSeq™-C0251 anti-human Hashtag 1 antibody	BioLegend	Cat# 394661
TotalSeq™-C0252 anti-human Hashtag 2 antibody	BioLegend	Cat# 394663
TotalSeq™-C0253 anti-human Hashtag 3 antibody	BioLegend	Cat# 394665
TotalSeq™-C0254 anti-human Hashtag 4 antibody	BioLegend	Cat# 394667
TotalSeq™-C0255 anti-human Hashtag 5 antibody	BioLegend	Cat# 394669
TotalSeq™-C0256 anti-human Hashtag 6 antibody	BioLegend	Cat# 394671
TotalSeq™-C0257 anti-human Hashtag 7 antibody	BioLegend	Cat# 394673
TotalSeq™-C0258 anti-human Hashtag 8 antibody	BioLegend	Cat# 394675
TotalSeq™-C0259 anti-human Hashtag 9 antibody	BioLegend	Cat# 394677

### Datasets

#### Stoeckius-2018

A single-cell dataset from human peripheral blood mononuclear cells has been previously published [8]. This dataset is comprised of eight individual donors that are uniquely labeled by eight cell hashtags. This dataset can be accessed from Gene Expression Omnibus (GEO) repository under accession number GSE108313.

#### 3V007

A novel single-cell dataset was generated for this paper. In this dataset, mRNA, B cell receptor (BCR) repertoire, surface protein expression (CD27 and CD79b), and antigen-CD79b ratios of 14 antigen-probes, including HA proteins of several endemic influenza strains, were measured. Two groups of cells (from the same donor), antigen-specific B cells (influenza HA-specific), and carrier cells (T cells and B cells) were uniquely labeled using two cell hashtags, hashtag1 and hashtag2, respectively. This dataset has been uploaded to GEO (see the data availability statement for details).

#### S414

A novel single-cell dataset was generated for this paper. In this dataset, mRNA, BCR repertoire, surface protein expression (CD27 and CD79b), and predicted binding specificity against eight antigen probes, including HA proteins of several endemic influenza strains, were measured. Four groups of cells (from the same donor) were uniquely labeled by four cell hashtags, hashtag3, hashtag4, hashtag5, and hashtag6. We split the carrier cells (T cells with a few B cells) into two groups, labeled them with hashtag3 and hashtag4; and split the antigen-specific B cells into two groups, labeling them with hashtag5 and hashtag6. This dataset has been uploaded to GEO (see the Data availability statement for details).

#### R125

This dataset has been previously published [26]. In this dataset, mRNA, BCR repertoire and predicted binding specificity against 17 antigen-probes, including Spike, NP, ORF8, and RBD proteins of the endemic and pandemic COVID strains, HA proteins of the influenza virus and interferon alpha and omega, were measured. Cells from three individual human donors were uniquely labeled by three cell hashtags: hashtag1-R1, hashtag2-R2, and hashtag3-R5. This dataset (R125) is available from Mendeley Data: https://doi.org/10.17632/3jdywv5jrv.3.

#### R6

This dataset has been previously published [26]. In this dataset, mRNA, BCR repertoire, and predicted binding specificity against 17 antigen probes, including Spike, NP, ORF8, and RBD proteins of the endemic and pandemic COVID strains, HA proteins of the influenza virus and interferon alpha and omega, were measured. Cells from two time points of an individual human donor were uniquely labeled by two cell hashtags: hashtag3-early and hashtag4-late. This dataset (R6) is available from Mendeley Data: https://doi.org/10.17632/3jdywv5jrv.3.

#### 9pool-CA

A novel single-cell dataset was generated for this paper (CA is short for carrier). In this dataset, we sorted B cells and T cells from nine subjects and pooled them together. mRNA and surface protein expression panels were measured. Cells from nine individual human donors were uniquely labeled by nine cell hashtags: hashtag1–hashtag9. This dataset has been uploaded to GEO (see the Data availability statement for details).

#### 9pool-AS

A novel single-cell dataset was generated for this paper (AS is short for antigen-specific). In this dataset, we sorted antigen-specific B cells from nine human donors (as in dataset 8pool) and pooled them together. mRNA, BCR repertoire, surface protein expression (CD27 and CD79b), and predicted binding specificity against 18 antigen probes, including HA proteins of several endemic influenza strains, were measured. This dataset has been uploaded to GEO (see the Data availability statement for details).

#### 8pool-AS

A novel single-cell dataset was generated for this paper. In this dataset, we sorted antigen-specific B cells from eight subjects and pooled them together. mRNA, BCR repertoire, surface protein expression (CD27 and CD79b), and predicted binding specificity against several antigen probes, including HA proteins of several endemic influenza strains, were measured. This dataset has been uploaded to GEO (see the Data availability statement for details).

#### 8pool-CA

A novel single-cell dataset was generated for this paper. In this dataset, we sorted B cells and T cells from eight subjects (as in Dataset 8) and pooled them together. mRNA and surface protein expression panels were measured. Cells from eight individual human donors were uniquely labeled by eight cell hashtags: hashtag1–hashtag8. This dataset has been uploaded to GEO (see the Data availability statement for details).

### Cell hashing demultiplexing using HTOreader

We developed and introduced an improved demultiplexing approach for single-cell cell hashing, called HTOreader. To accurately determine the hashtag identity for each individual cell, we developed a cutoff calling method that precisely distinguishes true positive from background. Specifically, the distributions of normalized counts for each hashtag were first fitted into two Gaussian distributions, representing background and true positive groups. Then a cutoff value that distinguishes the two groups was calculated based on the means and standard divisions of these two Gaussian distributions. Finally, the identity of each individual cell was determined according to the hashtags they were positive for.

#### Data normalization

Two normalization methods—CLR and Log (log1*p*) normalization—are available. CLR method is more common in the normalization of CITE-seq protein expression and hashtags [22, 27]. For a given raw count vector $W$ of a hashtag, the CLR normalization will be:


$$ \mathrm{CLR}(W)=\left[\log \frac{w_1}{g(w)},\log \frac{w_2}{g(w)},\dots, \log \frac{w_n}{g(w)}\right] $$

where $n$ is the length of the vector $W$ and $g(w)={\left({\prod}_{i=1}^n{w}_i\right)}^{1/n}$ denotes the geometric mean of $W$.

The conventional log normalization also works well in some datasets. We use $\log 1p$ function ($\log 1p(N)=\log \left(N+1\right)$) to avoid the undefined log(0). For a given raw counts vector $W$ of a hashtag, the log normalization will be:


$$ \mathrm{Log}(W)=\left[\log \left({w}_1+1\right),\log \left({w}_2+1\right),\dots, \log \left({w}_n+1\right)\right]. $$

In datasets that incorporated a large number of hashtags in a single experiment (e.g. *N* > 4), we noted that for each hashtag signal, the “negative” peak significantly outweighs the “positive” peak. This is because the true positive cells for the current signal typically constitute 1/*N* of the entire population. Consequently, the two normal distributions fitted on the original data may be inaccurate. To address this, we perform a sampling for cells with low signals prior to model fitting when hashtag number *N* > 4. During the sampling phase, HTOreader employs an empirical cutoff to approximately identify low signals (1.5 for CLR and 3 for the log normalization method by default). We then arrange all cells with low signals in ascending order and select every *N*/2 cells (if *N*/2 is not an integer, use the floor value). Let NS denote a set of normalized signals and the negative signals and positive signals are two subsets of NS:


$$ {\mathrm{NS}}_{\mathrm{neg}}=\left\{x\in \mathrm{NS}|x<c\right\} $$

$$ {\mathrm{NS}}_{\mathrm{pos}}=\left\{x\in \mathrm{NS}|x\ge c\right\} $$

where $c$ is the cutoff. Then, we sort the ${\mathrm{NS}}_{\mathrm{neg}}$ in ascending order as ${\mathrm{NS}}_{\mathrm{neg}}^{\prime }$. Then, the sampled subset:


$$\begin{array}{l} {\mathrm{NS}}_{\mathrm{sample}}=\left\{{x}_1,{x}_{1+\theta },{x}_{1+2\theta },\dots, {x}_{1+ k\theta}\right\},k=1,2,3,\dots \mathrm{and}\\ 1+ k\theta <\mathrm{Card}\left({\mathrm{NS}}_{\mathrm{neg}}^{\prime}\right) \end{array}$$

where $\theta =\mathrm{floor}\left(N/2\right)$ and $x\in{\mathrm{NS}}_{\mathrm{neg}}^{\prime }$. The reshaped set will be:


$$ {\mathrm{NS}}_{\mathrm{reshape}}={\mathrm{NS}}_{\mathrm{sample}}\cup{\mathrm{NS}}_{\mathrm{pos}} .$$

Following this, we amalgamate the low signal sampling with all high signals as the reshaped data for the subsequent model fitting.

#### Mixture modeling

Mixture modeling has been extensively used in single-cell data pre-processing, such as to estimate drop-out rates or determine effective sequencing depth and amplification noise [28, 29]. We adopted a mixture modeling approach implemented in the Flexmix package to fit two Gaussian distributions from a vector of normalized hashtag counts [30]. In this step, we fit the normalized data of each hashtag into two Gaussian distributions indicating one positive group, representing background and true positive groups, and calculate the means and standard deviations of these two groups, respectively.

#### Cutoff determination

For two Gaussian distributions $N\left({\mu}_1,{\sigma}_1^2\right)$ and $N\left({\mu}_2,{\sigma}_2^2\right)$, ${\mu}_1<{\mu}_2$, we determine the cutoff to distinguish true positive and background using the following equation:


$$ \mathrm{Cutoff}={\mu}_1+\frac{\sqrt[n]{\sigma_1}}{\sqrt[n]{\sigma_1}+\sqrt[n]{\sigma_2}}\left({\mu}_2-{\mu}_1\right) $$

where $n$ is the rank of the model and the recommended rank is 2 in most cases. To mitigate the risk of inaccurate cutoff calls, we introduced an additional validation step in HTOreader. If the selected threshold falls below a predefined empirical limit (defaulting to 1.5 for CLR and 3 for the log normalization method), it is automatically adjusted to the global minimum value based on the linear approximated density of the normalized cell hashing signal between the means of the two fitted distributions.

#### Sample identity assignment using cell hashing

We assign cells’ sample identities based on the binding status of each hashtag [22]. If a cell is deemed positive for only one hashtag, it will be labeled as a singlet for that corresponding hashtag; if it’s deemed positive for multiple hashtags, it will be labeled as a doublet; if it’s deemed background for all hashtags, it will be labeled as negative. Sample identities for every cell labeled as singlet will be assigned according to their hashtag identities.

### Genomic signature demultiplexing

Genomic signature demultiplexing is an essential part of this hybrid strategy, and to date, many computational methods are available, for example, demuxlet and Souporcell. In this paper, we applied Souporcell, which has been widely used in the community, as part of our strategy to demonstrate the effectiveness of this workflow. As most SNP-based demultiplexing methods do, Souporcell aligns all short reads against a reference genome to determine SNPs for each cell and then groups cells into multiple clusters according to their genotypes (SNP signatures) using an unsupervised learning algorithm. The number of genotype clusters is pre-defined by users according to the number of subjects in the pooled sample. For a sample pooled from *N* subjects (individual human donors), there will be *N* + 2 distinct genotype clusters identified: one ‘doublet’ cluster containing cells that fit with more than one genotypes and one ‘negative’ cluster containing cells whose SNP signatures are not sufficient and therefore cannot be fit into any genotype, and *N* singlet clusters indicating cells from *N* individual donors.

### Sample identity assignment in hybrid demultiplexing

First, we correlate results from both cell hashing demultiplexing and SNP-based demultiplexing to reveal the sample identity of genotype clusters. For each genotype group, the highest-correlated cell hashing group represents its sample identity (Supplementary Fig. S3A). Then, focusing on cells that are assigned as singlets by both methods (highlighted by red border in Supplementary Fig. S3A), we define the number of cells that received consistent assignment from both methods as N1 (highlighted by green background) and the number of cells that received inconsistent assignment from both methods as N2 (highlighted by orange background). So the convergence score is defined as:


$$ C=\frac{N_1}{N_1+{N}_2}. $$

If the convergence score exceeds a pre-defined threshold (a number between 0 and 1, with a default value of 0.7), as seen in 8pool-CA (convergence score = 0.99), we deem the experiment quality to be satisfactory. Subsequently, sample labels were assigned to all cells based on their assignments from both methods (Supplementary Fig. S3B). Cells receiving consistent labels from both methods were assigned the consistent label as their sample label. For cells receiving inconsistent labels, there’re three cases:

- Case 1: cells that are assigned as ‘doublet’ or “negative” by cell hashing methods but are assigned as “singlet” by the SNP-based method. Labels of these cells will be assigned as their genotype labels because they are “false doublet” or “false negative” cells due to the hashtag contamination or failure.
- Case 2: cells that are assigned as “singlet” by cell hashing methods but are assigned as “doublet” or “unassigned” by the SNP-based method. Labels of these cells will be assigned as “unassigned” since current data are insufficient to infer their sample identification with high confidence.
- Case 3: cells were assigned as singlet by both methods; however, the sample identities assigned by the two methods are inconsistent. Labels of these cells will be assigned as “unassigned” since current data is insufficient to infer their sample identification with high confidence.

If the convergence score falls below the threshold, as observed in 9pool-CA (score = 0.1 and 0.55 for Supplementary Tables S14 and S15, respectively), it suggests a potential high doublet rate or disrupted genetic background among samples in the current dataset. Cell labels will then be assigned based on their cell hashing labels. Additionally, a warning message will be displayed to alert the user that the demultiplexing results may not be accurate and that the data quality requires the user’s attention and biological knowledge.

### Cell hashing demultiplexing comparison

We conducted demultiplexing using popular existing methods, including MULTI-seq, GMM-Demux, DropletUtils, BFF_raw, and BFF_cluster, utilizing the R package cellhashR (version 1.0.3) in R 4.2.2. Additionally, we ran HTOreader using the R package HTOreader (version 0.1.0) and HTOdemux using the R package Seurat (version 4.3.0.1) in R 4.2.2. For cellhashR and HTOreader, all parameters were set to default. For Seurat, the positive quantile was set to 0.99, with the remaining parameters set to their default values.

When testing methods under different parameter settings, we conducted MULTI-seq and BFF_cluster using cellhashR package (version 1.0.3) in R 4.2.2. We ran HTOdemux using R package Seurat (version 4.3.0.1) in R 4.2.2. We conducted GMM-Demux using GMM_Demux (version 0.2.2.3) (details in Supplementary Data).

We used the following measurements to evaluate the performance of all tested methods: singlet rate, accuracy, recall, precision, and F1-score. The singlet rate is defined as the percentage of identified singlets in all cells. For benchmark dataset Stoeckius-2018, we utilized ground truth labels generated using a SNP-based demultiplexing method, scSplit (see Supplementary Data for details). For in-house datasets 8pool-CA and R125, we generated ground truth labels using Souporcell. We classify singlets correctly identified as singlets as true positives (TP), doublets/negative cells accurately identified as doublets/negative cells as true negatives (TN), doublets/negative cells misclassified as singlets as false positives (FP) and singlets erroneously labeled as doublets/negative as false negatives (FN). Then, we define:


$$ \mathrm{Accuracy}=\frac{\mathrm{TP}+\mathrm{TN}}{\mathrm{TP}+\mathrm{TN}+\mathrm{FP}+\mathrm{FN}} $$

$$ \mathrm{Recall}=\frac{\mathrm{TP}}{\mathrm{TP}+\mathrm{FN}} $$

$$ \mathrm{Precision}=\frac{\mathrm{TP}}{\mathrm{TP}+\mathrm{FP}} $$

$$ F1=2\ast \frac{\mathrm{Precision}\ast \mathrm{Recall}}{\mathrm{Precision}+\mathrm{Recall}}=\frac{2\ast \mathrm{TP}}{2\ast \mathrm{TP}+\mathrm{FP}+\mathrm{FN}}. $$

### Flow cytometry staining and cell sorting

Flow staining and cell sorting were performed as previously described [26]. Briefly, human PBMCs were thawed in 10% FBS RPMI-1640 medium and enriched by negative selection using a pan-B cell isolation kit according the manufacturer’s instruction (StemCell, Cat#. 19554) prior to staining with the following antibodies and fluorescent oligonucleotide-labeled streptavidin-antigen tetramers (Biolegend): anti-huCD19-PE-Cy7, anti-huCD3-BB515, anti-huCD4-BB515, anti-huIgD-BB515, TotalSeq-C anti-human hashtag antibodies, antigen-PE or-APC and at 4° for 30 min. Cells were subsequently washed three times with 2% FBS/PBS buffer supplemented with 2 mM D-biotin. Finally, cells were adjusted at a maximum of 2 million cells per ml in washing buffer, stained with DAPI and subjected to sorting by either MACSQuantTyto (Miltenyi) or BD Melody (BD). Cells that were viable/CD19^+^/antigen-PE^+^ and antigen-APC^+^ or viable/CD4^+^ were sorted for downstream 10X Genomics processing.

### 10X genomics libraries construction and next generation sequencing

5′ gene expression, VDJ, and surface protein feature libraries were prepared using the 10X genomics platform as per the manufacturer’s instructions [Chromium Next GEM Single Cell 5′ (HT) Reagent Kits v2 (Dual Index)]. Three libraries were quantified by real-time quantitative PCR using KAPA Library Quantification Kits (Roche) and pooled at recommended ratio and sequenced using NextSeq1000 (Illumina) with 26 cycles for read 1, 10 cycles for i7/i5 index and 150 cycles for read 2.

## Results

### Overview of the hybrid demultiplexing strategy

**Figure 1:**
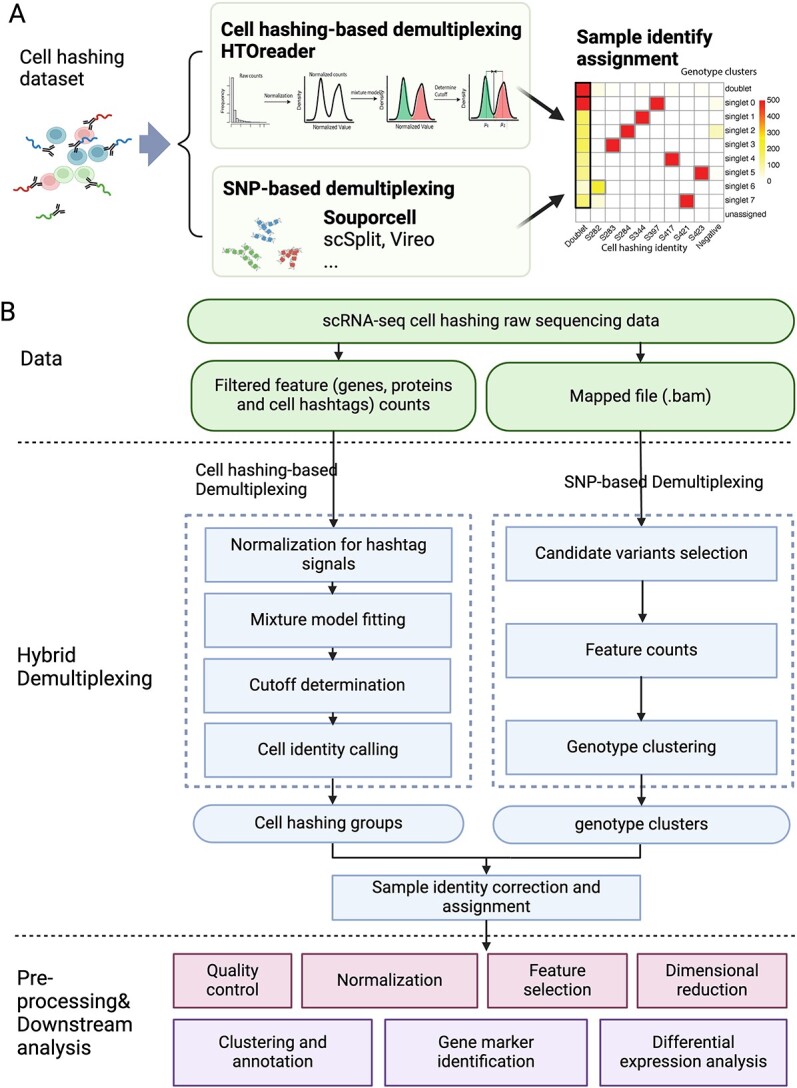
The architecture of hybrid demultiplexing strategy workflow for cell hashing data. (A) Graphical demonstration of the principle of hybrid demultiplexing. (B) Workflow and crucial components of hybrid demultiplexing strategy in conventional single-cell RNA-seq analysis. HTOreader was developed for cell-hashing-based demultiplexing and an existing tool Souporcell is the default SNP-based demultiplexing tool. This workflow is also compatible with other existing methods as demonstrated in the figure. Figure is created with BioRender.com.

The hybrid demultiplexing strategy addresses challenges in cell hashing experiments and existing demultiplexing methods, aiming to deliver accurate results across diverse experimental configurations. It incorporates two parallel demultiplexing solutions, cell-hashing-based demultiplexing and SNP-based demultiplexing, enhancing performance in a multi-modal fashion (Fig. 1A). Notably, the cell-hashing-based solution is sensitive to staining quality and experimental configurations, while the SNP-based solution consistently achieves high accuracy but lacks access to sample identity without additional experimental efforts. The hybrid strategy integrates results from both solutions, as illustrated in Figure 1A, allows mutual validation and reveals cell identities in an accurate and reliable manner.

The workflow for hybrid demultiplexing takes cell hashtag feature counts and raw sequencing reads as inputs for hashing- and SNP-based solutions, respectively. Downstream, pre-processing occurs in the form of HTOreader, an improved cut-off calling algorithm for cell hashing distributions, and Souporcell, an unsupervised clustering algorithm based on SNP variants (Fig. 1B) [10]. We developed HTOreader to consistently generate accurate hashtag threshold results for a variety of real-world datasets through a streamlined pipeline consisting of four steps (Fig. 1B). First, the raw counts of each individual hashtag are normalized. Next, the normalized counts are fit into two Gaussian distributions via a mixture model, representing background and true positive groups. Importantly, for datasets with more than four hashtags, the normalized data will be sampled before model fitting to avoid the dominance of negative signals. Subsequently, the optimal cutoff of each hashtag that distinguishes the two inferred distributions is determined. Finally, the sample identity of each cell is assigned based on its hashtag profile (Fig. 1B). For SNP-based demultiplexing, we employed Souporcell, which is comprised of three major steps: feature (genetic variant; SNP) selection, counting cell alleles and genotype-based clustering [10]. As the final step, the hybrid strategy correlates demultiplexing results from both methods and then reveals the sample identities for each cell. Singlet groups from the two methods are combined to unveil the sample identity of genotype clusters. Additionally, a convergence score was generated to assess the demultiplexing quality and identify potential errors. Subsequently, sample labels were assigned to all cells based on their assignments from both methods (see Methods for details).

Importantly, the hybrid strategy is flexible with existing demultiplexing methods, enabling users to choose preferred algorithms for both cell hashing and genetic variants (Fig. 1A). However, it requires diverse genetic backgrounds for accurate genotype cluster generation in pooled samples, making it unsuitable for experiments that share the same genetic background (e.g. genetically homogenous animal models). Careful consideration of sample pooling and demultiplexing solutions during experimental design ensures accuracy and maximizes performance. A tree diagram is provided to guide users in selecting demultiplexing solutions based on their experimental configurations (Supplementary Fig. S1).

### HTOreader, a demultiplexing tool to improve hashtag threshold determination in real-world datasets

**Figure 2:**
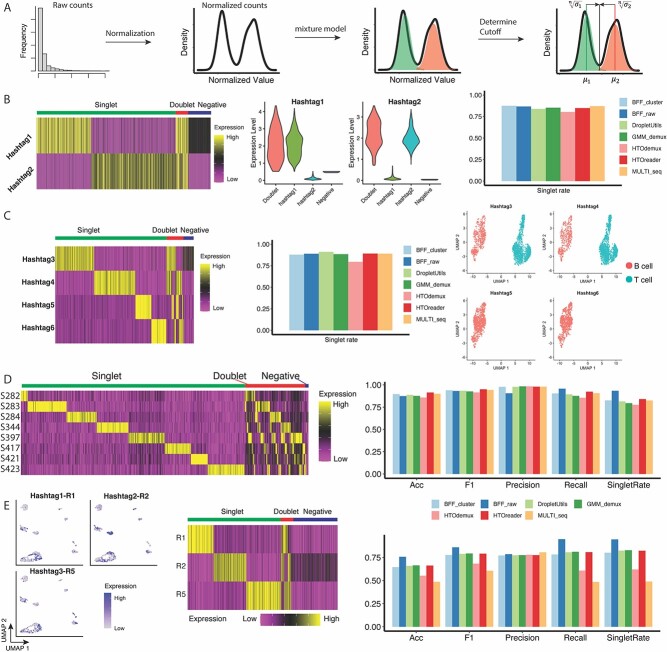
HTOreader achieves high accuracy and increases cell recovery and robustness across real-world datasets. (A) The workflow of HTOreader to determine proper cutoff for each cell hashtag. (B) Demultiplexing on 3V007. Left: expression heatmap of both hashtags in 3V007. Center: violin plot of expression of both hashtags on HTOreader groups. Right: Singlet rates of all seven tested methods on 3V007. (C) Demultiplexing on S414. Left: expression heatmap of all hashtags in S414; center: singlet rates of all seven tested methods on S414; right: cells on a UMAP plot grouped by HTOreader demultiplexing and colored by cell types. (D) Demultiplexing on 8pool-CA. Left: expression of eight cell hashtags on dataset 8pool-CA. Singlet, doublet, and negative groups are indicated at the top of the heatmap. Right: performance of seven tested methods on 8pool-CA. (E) Demultiplexing on R125. Left: expression of three hashtags on the UMAP embedding of dataset4. Center: expression heatmap of all hashtags. Right: Performance of seven tested methods on R125.

As mentioned above, current demultiplexing methods for cell hashing can perform poorly in real-world datasets with many hashtags, imbalanced sample sizes or highly variable hashtag distributions. Here, we developed HTOreader, aiming to improve the accuracy and singlet rate of cell-hashing-based demultiplexing in various real-world scenarios (Fig. 2A). Unlike HTOdemux and MULTI_seq, which utilize heuristic algorithms, HTOreader independently handles each HTO to mitigate interference from imbalanced sample sizes. It aligns with existing methods in determining cutoffs from a bimodal distribution but employs a unique approach by determining an optimal cutoff using the global minimum value on the linear approximated density of normalized HTO counts between positive and negative groups, distinguishing it from other existing methods.

Below, we compare the performances between HTOreader and the established method, including MULTI-seq, HTOdemux, GMM-Demux, DropletUtils, and BFF, on both the benchmark (Stoeckius-2018) and several real-world datasets (3V007, S414, 8pool-CA, and R125; generated in-house). Among the five datasets analyzed, three (Stoeckius-2018, 8pool-CA and R125) possess benchmark labels derived from SNP-based methods. The benchmark labels for Stoeckius-2018 were obtained from a previous study and generated by scSplit, while those for 8pool-CA and R125 were produced using Souporcell (details in Supplementary Data) [10, 16]. These representative datasets cover a variety of experimental settings and issues in the real world, including extensive usage of CITE-seq reagents (8pool-CA, S414 and 3V007), an imbalanced sample size (8pool-CA), a high number of hashtags (Stoeckius-2018 and 8pool-CA), variable hashtag distributions (8pool-CA) and suboptimal hashtag staining quality (R125). In general, the selection of these datasets aimed to comprehensively assess method efficacy across a broad spectrum of real-world experimental challenges.

The Stoeckius-2018 dataset, featuring eight hashtags and balanced sample sizes, serves as a benchmark dataset with available ground truth labels [8, 9]. As expected, all tested methods exhibited comparable singlet rates, ranging from 80% to 83% on Stoeckius-2018 (Supplementary Table S1). Upon validation with the ground truth labels, all methods delivered high performance in terms of accuracy, recall and F1 score (Supplementary Table S2). The performance of tested methods varies considerably when applied to real-world datasets. 3V007 and S414 are both balanced datasets with cells from single donors labeled with two and four hashtags, respectively (Fig. 2B and C). For these two datasets, most methods achieved over 85% singlet rate, except for HTOdemux, which only reached 80% (Fig. 2B and C, Supplementary Tables S3 and S4). Interestingly, DropletUtils generated inconsistent results for 3V007 when compared with other methods and failed to identify any doublets, suggesting low accuracy for this program (Supplementary Table S3). In S414, antigen-specific B cells were labeled with hashtags 5 and 6, while carrier B and T cells were labeled with hashtags 3 and 4. We highlighted cells identified by HTOreader for each hashtag on a UMAP and found that the demultiplexing result is consistent with experimental setup (Fig. 2C). The 8pool-CA dataset comprises cells from eight human donors labeled with eight hashtags, with very few cells from donor S282 (Fig. 2D). We observed significantly weaker peak separations in the hashtag data from 8pool-CA compared to the benchmark Stoeckius-2018, regardless of the normalization methods (Supplementary Fig. S2B and C). For 8pool-CA, two BFF models (BFF_raw and BFF_cluster) failed to identify any cell from sample S282, while the other methods produced reasonable singlet rates, ranging from 77.62% to 83.99% (Supplementary Table S5). Validation against the ground truth showed that all methods achieved high precision (>98.0%), except for BFF_cluster (90.6%) due to the failure in identifying S282 (Supplementary Table S6). All other methods also achieved high accuracy, recall and F1 scores (Supplementary Table S6, Fig. 2D). R125 comprises cells from three human donors and exhibits poor hashtag staining quality in plasmablasts, which express a disproportionally high level of immunoglobulin genes (Fig. 2E). As a result, the performance of all tested methods declined for R125. In particular, singlet rates calculated by MULTI_seq (49.7%) and HTOdemux (62.3%) were significantly lower than other methods due to the incorrect assignment of a large fraction of cells into the negative group. Interestingly, BFF_cluster generated the highest singlet rate (94.5%) (Supplementary Table S7) for R125 and outperformed all other methods when validating against ground truth labels (Fig. 2E, Supplementary Table S8).

We also investigated the performance of current methods with various parameter configurations, concentrating on those with adjustable parameters. The results indicated that HTOdemux and BFF_cluster reached optimal performance using the recommended parameter settings on most datasets. In contrast, the optimal parameters for MULTI_seq varied across different datasets, yet they were generally close to the default values. Additionally, even when the recommended parameters were not ideal, the difference in performance was marginal. It is important to note that BFF_cluster was unable to identify sample S282 in the 8pool-CA dataset, and MULTI_seq encountered difficulties with dataset R125, regardless of the parameter settings employed (see Method for details).

In conclusion, our results show that despite the fact that most methods perform reasonably well on the datasets we tested, it is important to highlight that some existing methods suffer from certain issues and exhibit suboptimal performance when demultiplexing specific datasets. These issues include the inability to identify hashtags, incorrect doublet identification and low singlet rates, despite attempts at parameter tuning (Table 1). Notably, HTOreader demonstrates high accuracy and robustness across various benchmark and real-world datasets (Table 1), consistently delivering reliable results in cell hashing experiments.

**Table 1:** Comparisons of existing cell hashing demultiplexing methods on multiple real-world datasets

	Multi-seq	HTOdemux	GMM_Demux	BFF_raw	BFF_cluster	HTOreader	DropletUtils
Stoeckius-2018	√	√	√	√	√	√	√
3 V007	√	False negative	√	√	√	√	False negative
S414	√	√	√	√	√	√	√
8pool-CA	√	Low recovery rate	Low recovery rate	Misdetection of a hashtag	Misdetection of a hashtag	√	√
R125	Low recovery rate	Low recovery rate	√	√	√	√	√

### Hybrid demultiplexing improves cell recovery and cost-effectiveness for cell hashing

The problem of cell loss stemming from hashtag failures and false doublets cannot be addressed solely by enhancing cell hashing signal demultiplexing and warrants further investigation. To address these issues, we exploit the recent advances in SNP-based demultiplexing methods that cluster single cells based on their SNPs. Here, we propose a hybrid demultiplexing strategy that integrates demultiplexing results from cell hashing and genetic variant profiles (Fig. 1A). Using this hybrid strategy, sample identities of single cells that are poorly stained (negative) or contaminated by multiple hashtags (doublet) can be determined together with those singlets from the same genotype cluster. Notably, this hybrid strategy is compatible with existing cell-hashing-based (GMM-Demux, MULTI_seq, HTODemux, DropletUtils and BFF) and SNP-based (scSplit, Vireo and Souporcell) demultiplexing methods (Fig. 1A). In the following analysis, we use HTOreader and Souporcell as a representative combination.

**Figure 3:**
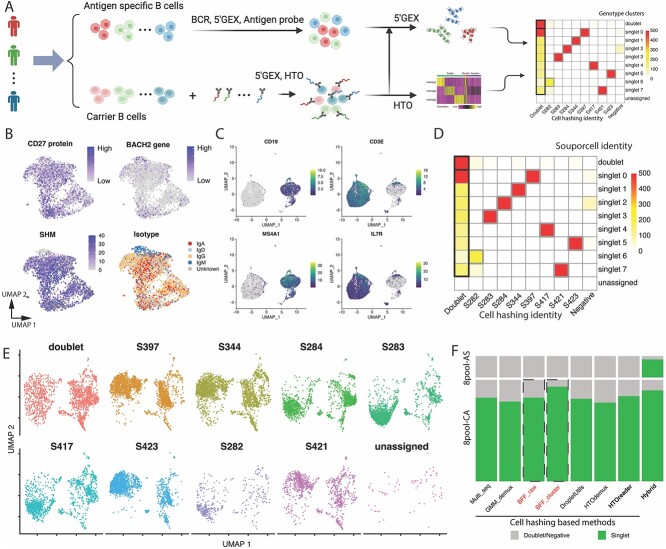
The hybrid demultiplexing strategy: enhancing cell recovery, robustness, and cost-effectiveness in a real-world immune cell dataset. (A) Depicts configurations and hybrid demultiplexing applied to datasets 8pool-CA and 8pool-AS. (B) UMAP visualization of expression of CD27 protein and BACH2 gene, somatic hypermutation (SHM) number of BCR heavy chain, and isotype of IG genes of all antigen-specific B cells (8pool-AS). (C) Expression of B cell (CD19 and MS4A1) and T cell (CD3E and IL7R) marker genes in carrier cells (dataset 8pool-CA). (D) A heatmap of correlation between cell hashing demultiplexing and SNP-based demultiplexing across all cells in dataset 8pool-CA. True doublets and false doublets were highlighted in thick black border, while genotype cluster-cell hashing pairs were highlighted in thick border. (E) Cells demultiplexed by this hybrid strategy of dataset 8pool-AS and dataset 8pool-CA are visualized on an integrated UMAP individually. (F) Demultiplexing results of different methods across both 8pool-CA and 8pool-AS datasets. Two BFF models were highlighted by red and dashed borders due to the failure of identifying sample S282 in dataset 8pool-CA. Panel A is created with BioRender.com.

To demonstrate its performance, we applied this hybrid strategy to a real-world dataset (8pool, Fig. 3A). In this dataset, a small aliquot of PBMCs from each of eight donors were stained with hashtag antibodies individually, and subsequently pooled together to sort carrier CD19+ B cells and CD4+ T cells (8pool-CA, Fig. 3C). Thereafter, all remaining PBMCs were pooled together to sort antigen-specific B cells (8pool-AS, Fig. 3B). We ran cells from 8pool-CA and 8pool-AS combined through Souporcell, and cells from 8pool-CA through HTOreader. Results showed that all hashtags were stained with high quality, and HTOreader divided all cells in 8pool-CA into 10 groups, including 10 821 cells from 8 singlet groups (84.0%), 1893 apparent doublets (14.7%) and 170 negative cells (1.3%) (Supplementary Table S5). In comparison, Souporcell divided all cells in 8pool into 10 genotype clusters. Among 12 884 cells from 8pool-CA, 11 550 cells were categorized into 8 singlet clusters (89.7%), 1326 cells into a doublet cluster (10.29%) and 8 cells into an unassigned cluster (0.06%) (Fig. 3D, Table 2). Using our hybrid strategy, the sample identities of eight singlet genotype clusters can be easily obtained by correlation with cell-hashing-based results (Table 2). 97.9% (10 597 from 10 821) of singlets identified by HTOreader were also registered as singlets in Souporcell, validating the accuracy of both methods (Fig. 3D). Within the extra singlets recovered by hybrid demultiplexing (5.2%), a small fraction (166 cells) was identified as negative, and the majority was identified as doublet (715 cells) by cell hashing, suggesting a high false doublet rate when using many hashtags (Fig. 3B, Table 2).

**Table 2:** Hybrid demultiplexing on dataset 8pool-CA and 8pool-AS. SNP demultiplexing method is Souporcell, cell hashing demultiplexing method is HTOreader

		SNP demultiplexing									
		Doublet	Singlet 0	Singlet 1	Singlet 2	Singlet 3	Singlet 4	Singlet 5	Singlet 6	Singlet 7	Unassigned
Cell hashing demultiplexing on Dataset 8pool-CA	Doublet	1173	166	80	88	103	70	82	51	75	5
	Negative	4	31	4	49	8	28	23	17	6	0
	S282	54	0	0	0	0	1	0	210	51	1
	S283	20	0	1	1	1748	0	0	1	0	0
	S284	9	0	0	1492	0	1	0	0	0	0
	S344	21	0	1528	0	1	2	0	8	0	0
	S397	8	2046	0	0	0	0	0	1	0	0
	S417	15	0	0	0	1	1153	1	2	0	2
	S421	4	0	0	0	0	0	0	0	776	0
	S423	18	0	0	0	0	0	1644	0	0	0
Dataset 8pool-AS		416	391	868	71	130	447	28	118	232	63

In addition to improving cell recovery and calling accuracy, this hybrid strategy can also decrease the experimental cost when processing samples with large numbers of cells. As we mentioned, cells from 8pool-AS and 8pool-CA shared the same donors and SNP profiles, so we ran Souporcell on all cells together to demultiplex cells from 8pool-AS that were not labeled with hashtags. Results showed that we identified 2285 cells in eight singlet clusters (82.67%), along with 416 apparent doublets (15.05%) and 63 negative cells (2.28%) (Table 2). Our hybrid strategy achieved an overall cell recovery rate of 88.03% (13 777 out of 15 650) with all cells from 8pool-AS and 8pool-CA combined (Fig. 3E and F, Table 2). Therefore, when processing samples in large scale using this hybrid strategy, only a small aliquot of cells from each donor (e.g. 100 cells per sample is sufficient for hybrid demultiplexing in practice) needs to be stained with hashtags to link donor identity with SNP clusters, and all cells can be efficiently demultiplexed through SNP-based clustering. This unique feature not only enhances experimental flexibility but also reduces the consumption of hashtag antibodies per sample without sacrificing performance. Together, compared to demultiplexing with cell hashing alone, our hybrid strategy demonstrates enhancements in both cell recovery rate and cost-effectiveness.

### Hybrid demultiplexing is more robust and reliable than unimodal methods

Another important strength of the hybrid strategy is its ability to deliver accurate results, largely independent of the hashtag staining quality or performance of cell hashing-based demultiplexing methods. For instance, regardless of the demultiplexing methods used in 8pool-CA, the hybrid strategy consistently correlated cell hashing singlet groups with genotype clusters and accurately determined the sample identity of almost 90% of cells (Table 2). In an extreme case where two BFF models failed to identify cells from sample S282, the hybrid strategy successfully correlated seven cell hashing groups identified by the BFF models with genotype clusters and revealed that a cluster of singlets represented cells from sample S282, as they were not labeled by any hashtags (Supplementary Tables S9 and S10). Therefore, the hybrid strategy permits up to one unlabeled sample [among *N* samples, (*N* − 1) being labeled with hashtags], either due to experimental design or hashtag failure. Unsurprisingly, for the other three methods that generated high quality results, our hybrid strategy also seamlessly correlated cell hashing singlet groups with genotype clusters (Supplementary Tables S11–S13).

**Figure 4:**
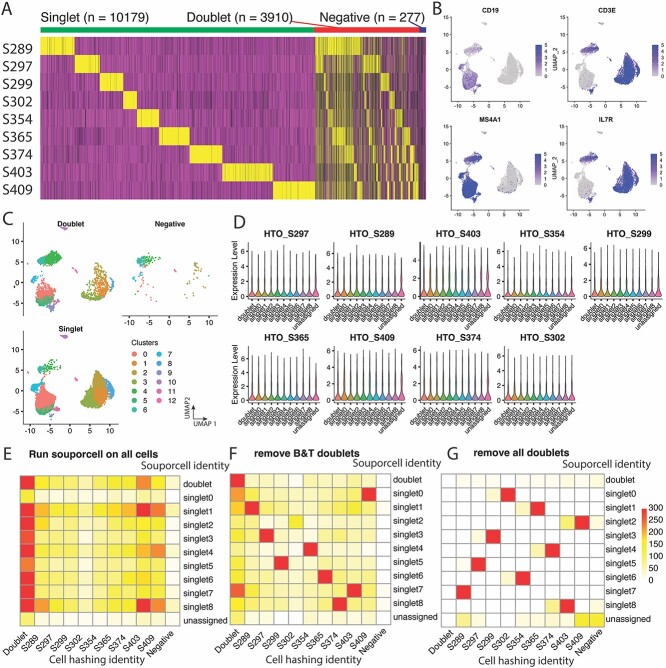
Hybrid demultiplexing strategy corrects potential error of SNP-based method. (A) Expression of nine cell hashtags on dataset 9pool-CA. Singlet, doublet, and negative groups are indicated on the top of the heatmap. (B) Expression of two B cell gene markers (CD19 and MS4A1) and two T cell gene markers (CD3E and IL7R) visualized on a UMAP embedding of carrier cells of dataset 9pool-CA. (C) Doublets, negative cells and singlets identified by cell hashing method are visualized on a UMAP individually. Cells are colored by transcriptome clusters. (D) Expression of nine hashtags on genotype clusters generated by SNP-based method. (E) A heatmap of correlation between cell hashing demultiplexing and SNP-based demultiplexing on all cells in dataset 9pool-CA. (F) A heatmap of correlation between cell hashing demultiplexing and SNP-based demultiplexing on all cells of dataset 9pool-CA after removing B&T doublets (cells that express both B and T cell markers). (G) A heatmap of correlation between cell hashing demultiplexing and SNP-based demultiplexing on all cells of dataset 9pool-CA after all potential doublets (cells that express more than one hashtag).

Although SNP-based methods are known to be highly accurate, we observed that Souporcell can generate incorrect genotype clusters in datasets with high true-doublet rates (Fig. 4A). When we applied our hybrid strategy to dataset 9pool-CA with T and B cells from nine donors, we observed a poor correlation (convergence score = 0.1) in singlets between HTOreader and Souporcell (Fig. 4D and E, Supplementary Table S14). With further analysis, we identified transcriptional clusters consisting of apparent true cell doublets expressing both B cell (CD19, MS4A1) and T cell (CD3E, IL7R) markers (Fig. 4B). Besides these B&T doublets, cell hashing signals revealed that there were more doublets in B cell and T cell clusters (Fig. 4C). Therefore, we suspected that the unexpectedly high proportion of true doublets in this dataset might interfere with the unsupervised clustering process of SNP-based methods. To test our hypothesis, we removed cell clusters expressing both B and T cell markers and observed an increased correlation (convergence score = 0.55, Fig. 4F, Supplementary Table S15). Then, we further removed all doublets assigned by cell hashing (Fig. 4D) and found that the convergence score reached 0.95 and demultiplexing results went back to normal (Fig. 4G, Supplementary Table S16). These results highlight the risk associated with SNP-based methods that use unsupervised clustering algorithms.

In conclusion, our results from dataset 8pool-CA show that the hybrid strategy leads to an enhanced singlet rate and accuracy compared to demultiplexing with cell hashing alone. Additionally, results from dataset 9pool-CA indicate that the hybrid strategy can identify incorrect genotype clustering generated by SNP-based methods, thereby highlighting potential experimental flaws. By integrating results from cell-hashing-based and SNP-based methods, the hybrid strategy allows the two methods to validate results from each other and correct potential errors, making it more robust and reliable than unimodal methods.

## Discussion

Over the last few years, sample super loading and demultiplexing strategies have been extensively studied to improve the quality, magnitude, and economy of single-cell experiments. Among them, cell hashing that derives from CITE-seq technology has been one of the most popular demultiplexing approaches due to its simplicity and compatibility with common sequencing platforms. However, the current cell hashing technique still has several disadvantages, including difficulties in distinguishing true positive from a background signal, unpredictable hashtag failure, high false doublet rates when using many hashtags and high reagent costs when staining large numbers of cells. Here, we developed an R package that includes an improved cell hashing demultiplexing tool, HTOreader and utilizes a hybrid demultiplexing strategy to integrate the results of cell hashing and SNP clustering, aiming to address the issues above.

One major caveat of cell hashing is the proper cutoff calling for cell hashing signals. Among existing cell-hashing demultiplexing methods, the mathematical models underlying different methods are based on various assumptions about the distribution of cell-hashing signals. When the actual distribution significantly deviates from these assumptions, the methods may fail, leading to serious consequences, such as misdetection of hashtags or inflated negative/doublet rate. Our findings indicate that existing methods faltered on certain datasets, and this was not rectifiable by adjusting parameters. Despite attempts to optimize performance through parameter tuning on multiple datasets, our results suggested that while tuning parameters can marginally improve performance, it is not sufficient to overcome failures on challenging datasets, which are likely due to significant deviations from the assumptions made by the mathematical models.

Another major challenge is that the cell recovery rate decreases as the number of hashtags increases, due to the accumulation of false doublets. In addition, the poor staining quality of certain hashtags that occur sporadically can further decrease cell recovery by increasing the negative rate. The SNP-based demultiplexing method is highly precise and capable of recovering up to 90% of cells. However, its utility is constrained by the need for distinct genetic backgrounds among samples and the additional expense of creating SNP references. Consequently, we developed a hybrid demultiplexing strategy that combines cell-hashing-based methods with SNP-based methods, allowing the strengths of each method to compensate for the other’s weaknesses and yield the most precise results. Our results showed that this hybrid strategy consistently increases cell recovery to ~90%, regardless of hashtag numbers or staining quality. This approach is extremely useful to simultaneously demultiplex multiple pooled samples from the same group of donors (such as longitudinal studies); only cells from one timepoint need to be hashtagged to obtain donor identities for cells from all timepoints (e.g. 9pool-CA + 9pool-AS, 8pool-CA + 8pool-AS). Additionally, our findings indicate that the hybrid strategy effectively addresses common errors in cell hashing demultiplexing, such as misidentifying hashtags and low singlet rates. Conversely, it also sensitively detects flaws in SNP-based demultiplexing, especially when cells lack genetic distinctiveness, thus providing a warning of potential data quality issues. Thus, in contrast to unimodal approaches, multimodal methods hold promise for yielding more precise and reliable demultiplexing results [20]. To conclude, our hybrid strategy provides a solution to improve the performance, robustness, and cost-effectiveness of single-modal cell hashing- and SNP-based methods.

Despite the advantages offered by the hybrid demultiplexing strategy, it still has several limitations. First, SNP-based demultiplexing only works with samples that have a distinct genetic background. As a result, it is not applicable in most animal studies or longitudinal studies with samples from the same donor. Additionally, the performance of the hybrid strategy is largely dependent on the quality of SNP-based clustering. Despite being more robust than cell-hashing-based methods, SNP-based clustering can still produce low-quality results due to multiple reasons, including cell populations with abnormal gene expression profiles (e.g. plasmablasts or dying cells), high true doublet rates, and poor transcriptome quality (e.g. insufficient reads or ambient RNA contamination). Thus, future efforts to improve the robustness of SNP-based clustering are necessary, which in turn will also enhance the performance of hybrid demultiplexing. Theoretically, this hybrid strategy is compatible with various barcode-based methods besides cell hashing; we plan to explore this potential by adapting it to other demultiplexing methods and examining performance using real-world datasets in future studies.

**Key Points**

- A hybrid demultiplexing strategy (cell hashing and genetic correlation) increases both cell recovery rate and calling accuracy.
- HTOreader accurately distinguishes true positives from background signal in datasets with (1) many hashtags or (2) imbalanced sample sizes.
- The hybrid approach enhances cost-effectiveness of cell hashing and consistently produces reliable demultiplexing results, regardless of hashtag staining quality.
- The hybrid strategy is compatible with existing single-cell experimental protocols and computational analysis tools.

## Supplementary Material

Supplementary_bbae254(1)

SupplementaryData_bbae254

## Data Availability

All single-cell datasets generated for this study have been uploaded into GEO database. The GEO accession number is GSE230810.

## Code availability

We have deposited the source code of the R package we developed in this study to GitHub: https://github.com/WilsonImmunologyLab/HTOreader. We have provided an example of hybrid demultiplexing on dataset 8pool-CA/AS and 9pool-CA as well. Both R markdown and HTML file can be found under https://github.com/WilsonImmunologyLab/HTOreader/tree/main/docs.

## References

[1] Tang F , Barbacioru C, Wang Y, et al. mRNA-Seq whole-transcriptome analysis of a single cell. Nat Methods 2009;6:377–82. 10.1038/nmeth.1315. 19349980

[2] Svensson V , Vento-Tormo R, Teichmann SA. Exponential scaling of single-cell RNA-seq in the past decade. Nat Protoc 2018;13:599–604. 29494575 10.1038/nprot.2017.149

[3] Stuart T , Satija R. Integrative single-cell analysis. Nat Rev Genet 2019;20:257–72. 10.1038/s41576-019-0093-7. 30696980

[4] Picelli S , Bjorklund AK, Faridani OR, et al. Smart-seq2 for sensitive full-length transcriptome profiling in single cells. Nat Methods 2013;10:1096–8. 10.1038/nmeth.2639. 24056875

[5] Klein AM , Mazutis L, Akartuna I, et al. Droplet barcoding for single-cell transcriptomics applied to embryonic stem cells. Cell 2015;161:1187–201. 10.1016/j.cell.2015.04.044. 26000487 PMC4441768

[6] Macosko EZ , Basu A, Satija R, et al. Highly parallel genome-wide expression profiling of individual cells using nanoliter droplets. Cell 2015;161:1202–14. 10.1016/j.cell.2015.05.002. 26000488 PMC4481139

[7] Cao J , Packer JS, Ramani V, et al. Comprehensive single-cell transcriptional profiling of a multicellular organism. Science 2017;357:661–7. 10.1126/science.aam8940. 28818938 PMC5894354

[8] Stoeckius M , Zheng S, Houck-Loomis B, et al. Cell hashing with barcoded antibodies enables multiplexing and doublet detection for single cell genomics. Genome Biol 2018;19:224. 10.1186/s13059-018-1603-1. 30567574 PMC6300015

[9] Kang HM , Subramaniam M, Targ S, et al. Multiplexed droplet single-cell RNA-sequencing using natural genetic variation. Nat Biotechnol 2018;36:89–94. 29227470 10.1038/nbt.4042 PMC5784859

[10] Heaton H , Talman AM, Knights A, et al. Souporcell: robust clustering of single-cell RNA-seq data by genotype without reference genotypes. Nat Methods 2020;17:615–20. 32366989 10.1038/s41592-020-0820-1 PMC7617080

[11] Cusanovich DA , Daza R, Adey A, et al. Multiplex single-cell profiling of chromatin accessibility by combinatorial cellular indexing. Science 2015;348:910–4. 25953818 10.1126/science.aab1601 PMC4836442

[12] Zhang Y , Xu S, Wen Z, et al. Sample-multiplexing approaches for single-cell sequencing. Cell Mol Life Sci 2022;79:466. 35927335 10.1007/s00018-022-04482-0 PMC11073057

[13] McGinnis CS , Patterson DM, Winkler J, et al. MULTI-seq: sample multiplexing for single-cell RNA sequencing using lipid-tagged indices. Nat Methods 2019;16:619–26. 31209384 10.1038/s41592-019-0433-8 PMC6837808

[14] Fang L , Li G, Sun Z, et al. CASB: a concanavalin A-based sample barcoding strategy for single-cell sequencing. Mol Syst Biol 2021;17:e10060. 33821571 10.15252/msb.202010060 PMC8022202

[15] Gehring J , Hwee Park J, Chen S, et al. Highly multiplexed single-cell RNA-seq by DNA oligonucleotide tagging of cellular proteins. Nat Biotechnol 2020;38:35–8. 31873215 10.1038/s41587-019-0372-z

[16] Xu J , Falconer C, Nguyen Q, et al. Genotype-free demultiplexing of pooled single-cell RNA-seq. Genome Biol 2019;20:1–12. 31856883 10.1186/s13059-019-1852-7 PMC6921391

[17] Huang Y , McCarthy DJ, Stegle O. Vireo: Bayesian demultiplexing of pooled single-cell RNA-seq data without genotype reference. Genome Biol 2019;20:1–12. 31836005 10.1186/s13059-019-1865-2 PMC6909514

[18] Shin D , Lee W, Lee JH, et al. Multiplexed single-cell RNA-seq via transient barcoding for simultaneous expression profiling of various drug perturbations. Sci Adv 2019;5:eaav2249. 10.1126/sciadv.aav2249. 31106268 PMC6520024

[19] Uzbas F , Opperer F, Sönmezer C, et al. BART-Seq: cost-effective massively parallelized targeted sequencing for genomics, transcriptomics, and single-cell analysis. Genome Biol 2019;20:1–16. 10.1186/s13059-019-1748-6. 31387612 PMC6683345

[20] Howitt G , Feng Y, Tobar L, et al. Benchmarking single-cell hashtag oligo demultiplexing methods. NAR Genom Bioinform 2023;5:lqad086. 10.1093/nargab/lqad086. 37829177 PMC10566318

[21] Stoeckius M , Hafemeister C, Stephenson W, et al. Simultaneous epitope and transcriptome measurement in single cells. Nat Methods 2017;14:865–8. 28759029 10.1038/nmeth.4380 PMC5669064

[22] Hao Y , Hao S, Andersen-Nissen E, et al. Integrated analysis of multimodal single-cell data. Cell 2021;184:3573–3587.e29. 10.1016/j.cell.2021.04.048. 34062119 PMC8238499

[23] Xin H , Lian Q, Jiang Y, et al. GMM-Demux: sample demultiplexing, multiplet detection, experiment planning, and novel cell-type verification in single cell sequencing. Genome Biol 2020;21:1–35. 10.1186/s13059-020-02084-2. PMC7393741 32731885

[24] Boggy GJ , McElfresh GW, Mahyari E, et al. BFF and cellhashR: analysis tools for accurate demultiplexing of cell hashing data. Bioinformatics 2022;38:2791–801. 35561167 10.1093/bioinformatics/btac213 PMC9113275

[25] Lun AT , Riesenfeld S, Andrews T, et al. EmptyDrops: distinguishing cells from empty droplets in droplet-based single-cell RNA sequencing data. Genome Biol 2019;20:63–9. 10.1186/s13059-019-1662-y. 30902100 PMC6431044

[26] Dugan HL , Stamper CT, Li L, et al. Profiling B cell immunodominance after SARS-CoV-2 infection reveals antibody evolution to non-neutralizing viral targets. Immunity 2021;54:1290–1303.e7. 10.1016/j.immuni.2021.05.001. 34022127 PMC8101792

[27] Aitchison J . The statistical analysis of compositional data. J R Stat Soc Series B Stat Methodology 1982;44:139–60. 10.1111/j.2517-6161.1982.tb01195.x.

[28] Fan J , Salathia N, Liu R, et al. Characterizing transcriptional heterogeneity through pathway and gene set overdispersion analysis. Nat Methods 2016;13:241–4. 26780092 10.1038/nmeth.3734 PMC4772672

[29] Kharchenko PV , Silberstein L, Scadden DT. Bayesian approach to single-cell differential expression analysis. Nat Methods 2014;11:740–2. 24836921 10.1038/nmeth.2967 PMC4112276

[30] Leisch F . FlexMix: a general framework for finite mixture models and latent glass regression in R. Journal of Statistical Software 2004;11:1–18. 10.18637/jss.v011.i08.

