# Supplementary material for: A hybrid demultiplexing strategy that improves performance and robustness of cell hashing
Source: Brief Bioinform. 2024 Jun 3;25(4):bbae254. doi: 10.1093/bib/bbae254 (PMC11145454; doi:10.1093/bib/bbae254)
Supplement: Supplementary_bbae254(1) [file supplementary_bbae254(1).docx]

**Limitations of cell hashing in performance and economy**

There are several factors that influence the performance of cell hashing, including cut-off calling accuracy, false doublet rate, and false negative rate. Our analysis on multiple real-world datasets reveal varying singlet rates: 81.67% (dataset Stoeckius-2018, Figure S4-A), 79.57% (dataset 3V007, Figure S4-B), 80.31% (dataset S414, Figure S4-C), 62.29% (dataset R125, Figure S4-D), and 30.56% (dataset R6, Figure S4-E). Firstly, even in the benchmark dataset with high-quality staining and a well-balanced distribution of cells in each sample (dataset Stoeckius-2018, Figure S3-A), we observed 16.33% of cells were identified as doublets, which include real doublets that consist of multiple individually-hashtagged cells sticking together, and false doublets that consist of single cells labeled with multiple hashtags. The inability to distinguish real doublets (which must be excluded from downstream analysis) from false doublets (which should be included if they pass quality control) results in substantial cell loss. Furthermore, 13.26%, 7.51%, and 31.04% of cells in datasets 3V007, S414, and R125 were identified as negative. Surprisingly, a large portion of these negative cells were singlets with low hashtag signal. We found that the built-in algorithm in Seurat is unable to accurately distinguish positive signal from background, and therefore incorrectly categorize these cells as negative (“negative” cells in Figure S4-B,C, and D have sufficient signal to hashtag1, hashtag3, and hashtag2, respectively). Finally, 68.98% of cells in dataset R6 were identified as negative due to poor staining quality of hashtags 3 and 4, rendering the longitudinal information for most cells in this dataset inaccessible (Figure S4-E). Under this situation, hashtag identities of cells that are not properly stained and only have background level of signal are lost unavoidably.

In addition to performance, experimental cost is another factor that limits the applicability of cell hashing. In the current protocol, all cells must be stained with a hashtag antibody labeled with a unique barcode that correlate with their sample identity. Consequently, the reagent cost is directly tied to the total number of cells that need to be stained, which can be expensive for massive datasets and large-scale studies. In conclusion, efforts that improve the cell recovery, accuracy, robustness, and cost-effectiveness of cell hashing is desperately needed for advancement of single-cell analysis.

**Ground truth label generation of benchmark datasets**

To compare the performance of testing methods on benchmark dataset Stoeckius-2018 and in-house dataset 8pool-CA and R125, we accessed the ground truth labels of cells using genetic-variant-based approaches.

For dataset Stoeckius-2018, the ground truth labels were accessed from a previous study (Xu et al, Genome Biology, 2019). The ground truth information was determined by a genetic-variant-based method (scSplit) and downloaded from <https://github.com/jon-xu/scSplit_paper_data>, more specifically, under folder “Table 4/PBMC”. Notably, there’re 15,583 cells in Stoeckius-2018 datasets. However, ground truth labels are only available for 7,931 barcodes. After removing barcodes that are not sequenced from GEX library, there are total 6,822 cells have both HTO data and ground truth labels. We then calculated the Accuracy, Recall, Precision and F1 from these 6,822 cells and list them in Table S2.

For dataset 8pool-CA and R125, the ground truth labels were determined by a genetic-variant-based method, Spouorcell (Heaton et al, Nature Methods, 2020). We feed the sorted BAM file from CellRanger output into Souporcell software and extracted the labels for all barcodes from its output. We then calculated the Accuracy, Recall, Precision and F1 using these labels.

Of note, we’re not able to generate the ground truth labels for dataset 3V007 and R414 because different samples in each dataset are originally from the same human donor and therefore cannot be distinguished by their SNP profiles. Therefore, we only accessed the singlet rate of tested methods on these two datasets.

**Performance assessment of existing methods under different parameters**

We conducted a performance comparison of various methods under different parameters to explore their limits. Among these methods, HTOreader and BFF_raw do not have adjustable parameters, except for the choice of normalization method (Log or CLR) in the case of HTOreader. DropletUtils offers an adjustable parameter "lower," but "by.rank" is recommended as an alternative to identify assumed empty droplets, with its value set to the number of cells. The remaining methods have one or more adjustable parameters, listed below along with their default or recommended values:

- BFF cluster: doublet_thresh = 0.05, neg_thresh = 0.05, dist_frac = 0.1
- GMM-demux: -t (a number between 0 to 1, default is 0.8)
- HTODemux: positive.quantile (a number between 0 to 1, recommended value is 0.99)
- Multi_seq: quantile (a number between 0 to 1, default value is 0.7).

We conducted performance tests on these methods under different parameters (Details in supplementary data). Firstly, we observed that GMM_Demux produced identical results across three tested datasets regardless of the parameter "t" value from 0.05 to 0.99. Variations in seed (seed = 1234 and 9999) or not providing a seed did not alter the results.

Secondly, the BFF_cluster method performed best with default parameter settings among 26 different configurations. However, it still failed to identify S282 in the 8pool-CA dataset. The running log indicated that rows of S282 were filtered out due to "low counts" by the BFF model.

The results from MULTI_seq indicated that the optimal value for the parameter "quantile" could vary across datasets. The best "quantile" values were found to be 0.7, 0.6, and 0.5 for the Stoeckius-2018, 8pool-CA, and R125 datasets, respectively. Although it achieved comparative performance on Stoeckius-2018 and 8pool-CA, it faltered on dataset R125 with low accuracy, recall, and F1 scores.

HTOdemux performed best on the Stoeckius-2018 and 8pool-CA datasets under default settings (positive.quantile = 0.99) and achieved optimal performance on R125 with positive.quantile = 0.8.

Our findings suggest that the recommended parameter settings for HTOdemux and BFF_cluster are effective for most datasets, whereas parameters of MULTI_seq need to be adjusted to suit different datasets. Notably, in cellhashR, multiple values between 0.2 and 0.95 are tested for MULTI_seq, and the best value is determined by ranking based on the singlet rate. This implementation highly increased the accuracy of the MULTI_seq method. Furthermore, although some methods achieved better performance through parameter adjustments, critical issues still persist on certain datasets.


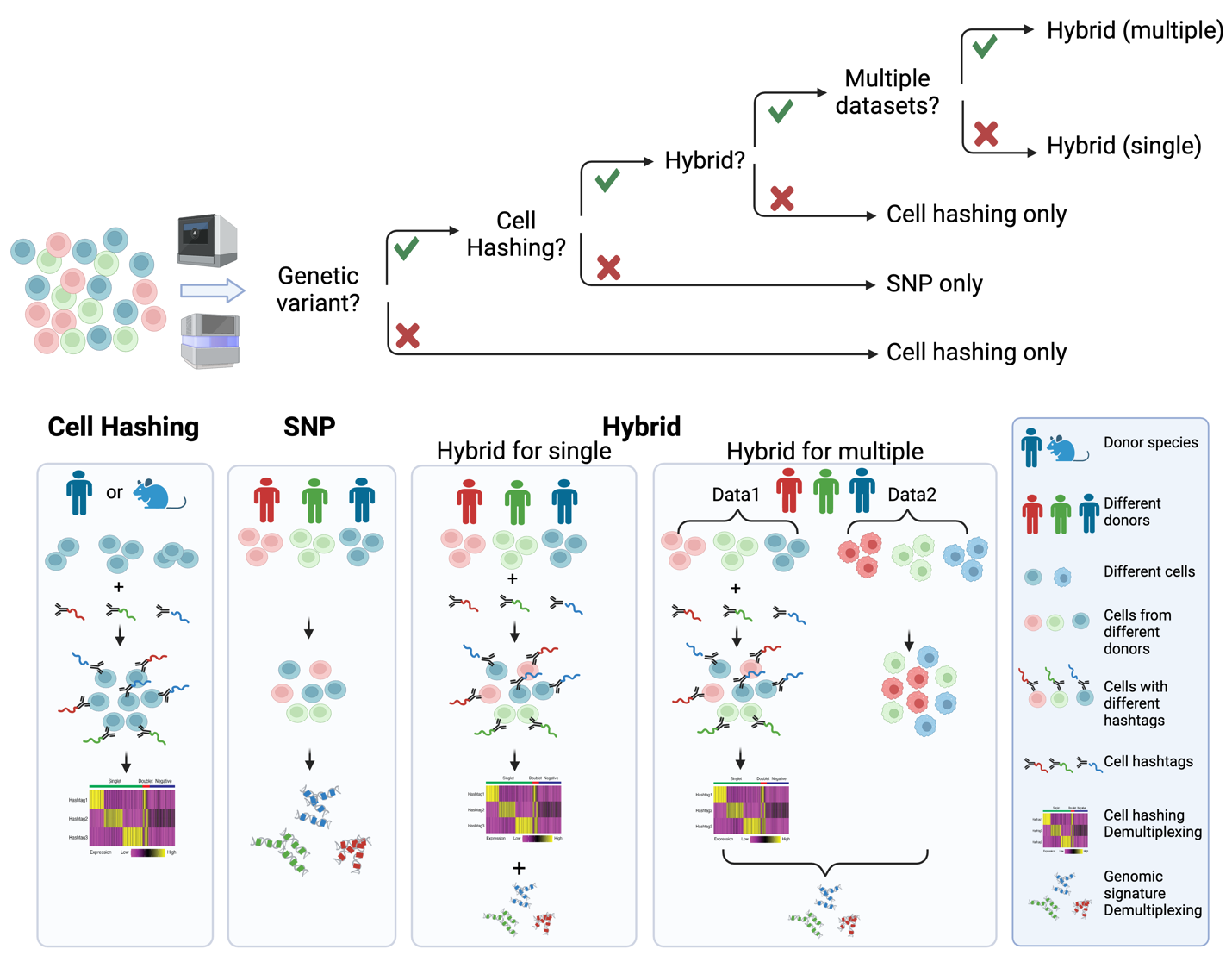


**Figure S1**. Graphical demonstration of scenarios of demultiplexing in cell hashing datasets: cell hashing-based demultiplexing, SNP-based demultiplexing and hybrid demultiplexing. A tree flow chart is provided for users to optimize demultiplexing methods aligned with their experimental parameters.


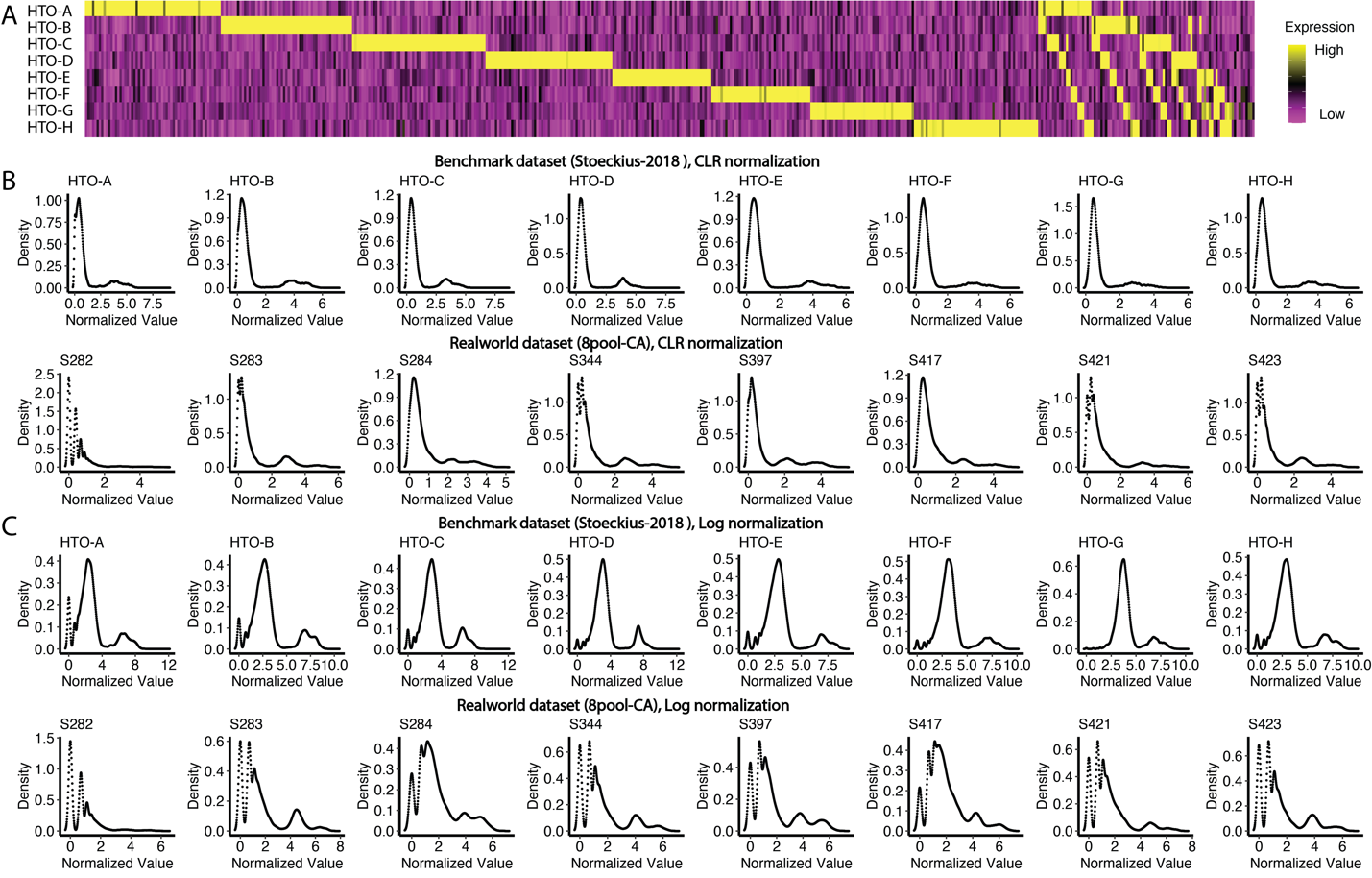


**Figure S2**. Comparison of cell hashing signals between a public benchmark dataset (Stoeckius-2018) and a real-world dataset (8pool-CA). (**A**) Expression heatmap of all eight hashtags in Stoeckius-2018. (**B**) Comparison of distribution of CLR normalized cell hashing signals between Stoeckius-2018 and 8pool-CA. (**C**) Comparison of distribution of Log normalized cell hashing signals between Stoeckius-2018 and 8pool-CA.

**Figure S3**. Sample identity assignment using hybrid demultiplexing. (**A**) Reveal sample identity of genotype groups and calculate the convergence score of two methods. (**B**) Final assignment of cell sample identities: consistent groups retain their labels, inconsistent-Case 1 groups are assigned genotype labels, inconsistent-case 2 and 3 croups are labeled 'Unassigned,' and inconsistent-unassigned groups are also labeled 'Unassigned.


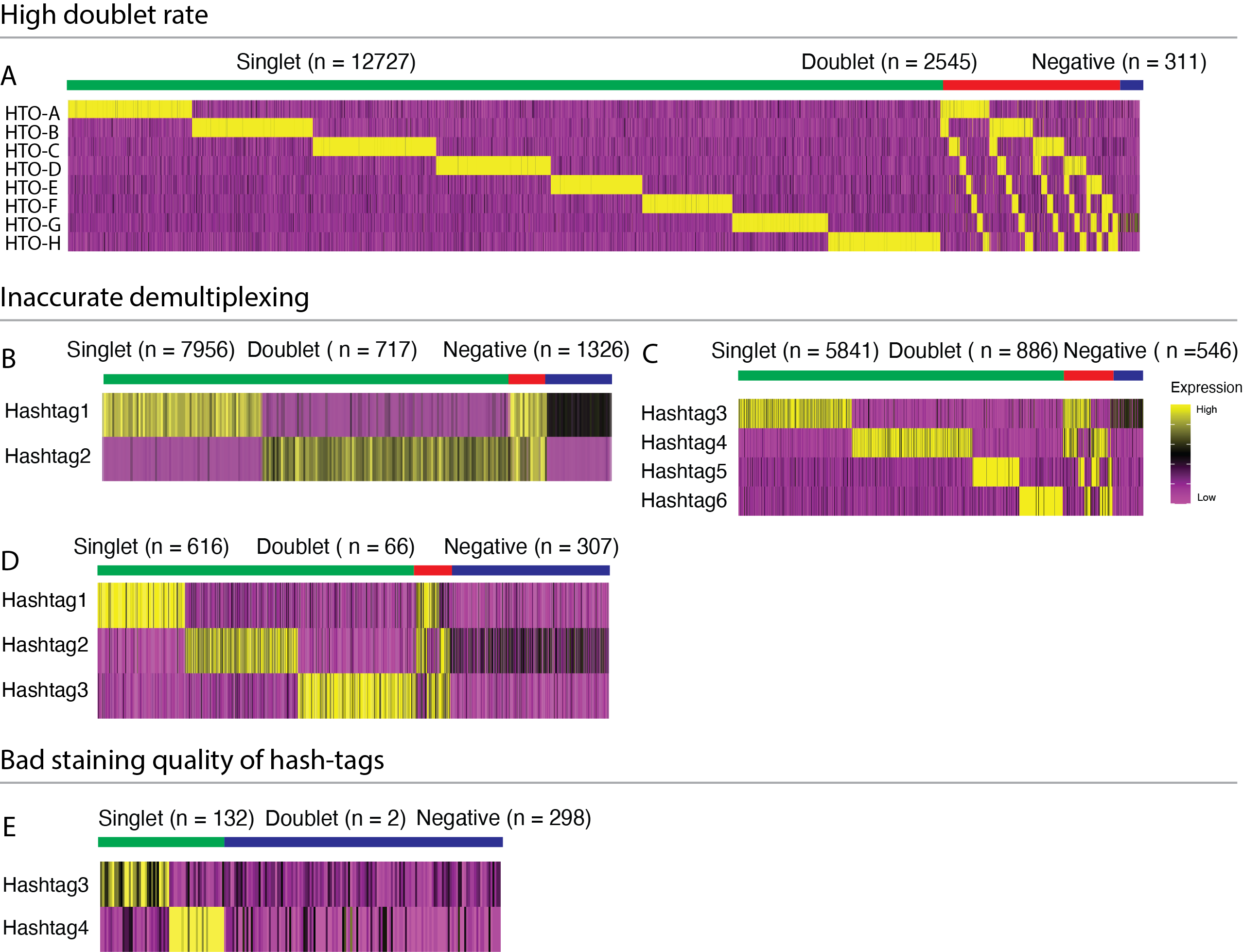


**Figure S4**. Limitations of cell hashing method revealed by real-world datasets. Expression of hashtags in datasets Stoeckius-2018, 3V007, S414, R125 and R6 are shown by heatmaps. Singlet, doublet, and negative groups were indicated by a color bar with number of cells in each group. Panels A – E share the same scale bar of heatmap to the right. Five datasets were grouped by three reasons of cell loss in cell hashing: high doublet rate, inaccurate demultiplexing due to insufficient hashtag signals, and bad staining quality of one or more hashtags. (**A**) Demultiplexing of dataset Stoeckius-2018. (**B**) Demultiplexing of dataset 3V007. (**C**) Demultiplexing of dataset S414. (**D**) Demultiplexing of dataset R125. (**E**) Demultiplexing of dataset R6.

**Table S1**. Cell hashing demultiplexing of dataset Stoeckius-2018 using existing methods. Number of total singlets and the singlet rate were highlighted in bold.

|  | **Multi-seq** | **GMM_demux** | **BFF_raw** | **BFF_cluster** | **DropletUtils** | **HTOreader** | **HTOdemux (Seurat)** |
| --- | --- | --- | --- | --- | --- | --- | --- |
| Doublet | 2172 | 2345 | 2427 | 2597 | 2378 | 2452 | 2545 |
| HTO_A | 1610 | 1679 | 1695 | 1660 | 1658 | 1712 | 1738 |
| HTO_B | 1783 | 1796 | 1807 | 1777 | 1809 | 1801 | 1800 |
| HTO_C | 1757 | 1747 | 1737 | 1710 | 1747 | 1739 | 1737 |
| HTO_D | 1607 | 1600 | 1596 | 1571 | 1612 | 1595 | 1597 |
| HTO_E | 1349 | 1349 | 1352 | 1327 | 1346 | 1348 | 1347 |
| HTO_F | 1296 | 1374 | 1390 | 1362 | 1394 | 1387 | 1373 |
| HTO_G | 1561 | 1654 | 1643 | 1611 | 1679 | 1644 | 1446 |
| HTO_H | 1628 | 1667 | 1698 | 1674 | 1692 | 1694 | 1689 |
| Negative | 820 | 372 | 238 | 294 | 268 | 211 | 311 |
| **singlet** | **12591** | **12866** | **12918** | **12692** | **12937** | **12920** | **12727** |
| **singlet rate** | **80.79%** | **82.56%** | **82.90%** | **81.45%** | **83.02%** | **82.91%** | **81.67%** |

**Table S2.** Performance of tested methods of dataset Stoeckius-2018 when validating with ground truth.

|  | **Acc** | **Recall** | **Precision** | **F1** | **singlet rate** |
| --- | --- | --- | --- | --- | --- |
| **Multi-seq** | 0.92670771 | 0.958318361 | 0.952159943 | 0.955229226 | 0.821166813 |
| **GMM_demux** | 0.952653181 | 0.980772686 | 0.961931618 | 0.971260788 | 0.831720903 |
| **BFF_raw** | 0.964819701 | 0.99047619 | 0.967187226 | 0.978693182 | 0.835385517 |
| **BFF_cluster** | 0.969070654 | 0.984189723 | 0.978039636 | 0.981105042 | 0.821020229 |
| **Dropletutils** | 0.957930226 | 0.98472871 | 0.964455393 | 0.974486621 | 0.833040164 |
| **HTOreader** | 0.966578716 | 0.991194969 | 0.96856892 | 0.979751332 | 0.834799179 |
| **HTOdemux(Seurat)** | 0.97566696 | 0.994610133 | 0.976022567 | 0.985228688 | 0.831427734 |

**Table S3**. Cell hashing demultiplexing of dataset 3V007 using existing methods. Number of total singlets and the singlet rate were highlighted in bold.

|  | **Multi-seq** | **GMM_demux** | **BFF_raw** | **BFF_cluster** | **DropletUtils** | **HTOreader** | **HTOdemux (Seurat)** |
| --- | --- | --- | --- | --- | --- | --- | --- |
| Doublet | 1032 | 1152 | 1101 | 1150 | 0 | 1070 | 717 |
| hashtag1 | 4299 | 4368 | 4371 | 4315 | 4326 | 4350 | 3059 |
| hashtag2 | 4598 | 4478 | 4521 | 4469 | 4793 | 4560 | 4897 |
| Negative | 70 | 1 | 6 | 65 | 880 | 19 | 1326 |
| **singlet** | **8897** | **8846** | **8892** | **8784** | **9119** | **8910** | **7956** |
| **singlet rate** | **88.98%** | **88.47%** | **88.93%** | **87.85%** | **91.20%** | **89.11%** | **79.57%** |

**Table S4**. Cell hashing demultiplexing of dataset S414 using existing methods. Number of total singlets and the singlet rate were highlighted in bold.

|  | **Multi-seq** | **GMM_demux** | **BFF_raw** | **BFF_cluster** | **DropletUtils** | **HTOreader** | **HTOdemux (Seurat)** |
| --- | --- | --- | --- | --- | --- | --- | --- |
| Doublet | 746 | 928 | 788 | 699 | 1015 | 967 | 886 |
| hashtag3 | 2619 | 2548 | 2606 | 2614 | 2520 | 2527 | 2060 |
| hashtag4 | 2151 | 2123 | 2136 | 2158 | 2051 | 2115 | 2153 |
| hashtag5 | 810 | 802 | 813 | 823 | 800 | 798 | 850 |
| hashtag6 | 745 | 725 | 751 | 766 | 733 | 723 | 778 |
| Negative | 202 | 147 | 179 | 213 | 154 | 143 | 546 |
| singlet | 6325 | 6198 | 6306 | 6361 | 6104 | 6163 | 5841 |
| singlet rate | 86.97% | 85.22% | 86.70% | 87.46% | 83.93% | 84.74% | 80.31% |

**Table S5**. Cell hashing demultiplexing of dataset 8pool-CA using existing methods. Number of total singlets and the singlet rate were highlighted in bold.

|  | **Multi-seq** | **GMM_demux** | **BFF_raw** | **BFF_cluster** | **DropletUtils** | **HTOreader** | **HTOdemux (Seurat)** |
| --- | --- | --- | --- | --- | --- | --- | --- |
| Doublet | 2103 | 2731 | 1673 | 456 | 1801 | 1893 | 2759 |
| Negative | 160 | 27 | 579 | 404 | 599 | 170 | 125 |
| S282 | 312 | 312 | 0 | 0 | 267 | 317 | 302 |
| S283 | 1738 | 1680 | 1786 | 2011 | 1769 | 1771 | 1698 |
| S284 | 1546 | 1480 | 1515 | 1713 | 1463 | 1502 | 1389 |
| S344 | 1548 | 1474 | 1585 | 1771 | 1512 | 1560 | 1476 |
| S397 | 1866 | 1669 | 2094 | 2380 | 1953 | 2055 | 1635 |
| S417 | 1196 | 1160 | 1179 | 1376 | 1184 | 1174 | 1148 |
| S421 | 780 | 720 | 794 | 890 | 791 | 780 | 727 |
| S423 | 1635 | 1631 | 1679 | 1883 | 1545 | 1662 | 1625 |
| **singlet** | **10621** | **10126** | **10632** | **12024** | **10484** | **10821** | **10000** |
| **singlet rate** | **82.44%** | **78.59%** | **82.52%** | **93.33%** | **81.37%** | **83.99%** | **77.62%** |

**Table S6.** Performance of tested methods of dataset 8pool-CA when validating with ground truth.

|  | **Acc** | **Recall** | **Precision** | **F1** | **singlet rate** |
| --- | --- | --- | --- | --- | --- |
| **Multi-seq** | 0.90018628 | 0.90654124 | 0.97994539 | 0.94181522 | 0.82435579 |
| **GMM_demux** | 0.87612543 | 0.87589208 | 0.9833887 | 0.92653287 | 0.79431853 |
| **BFF_raw** | 0.89622788 | 0.9040953 | 0.97799097 | 0.93959246 | 0.82520956 |
| **BFF_cluster** | 0.87387457 | 0.9566172 | 0.90593812 | 0.93058819 | 0.93325054 |
| **Dropletutils** | 0.88683639 | 0.89325549 | 0.97777566 | 0.93360656 | 0.81372245 |
| **HTOreader** | 0.91423471 | 0.92324447 | 0.97929951 | 0.95044621 | 0.83987892 |
| **HTOdemux(Seurat)** | 0.8576529 | 0.85535248 | 0.9828 | 0.91465798 | 0.77615647 |

**Table S7**. Cell hashing demultiplexing of dataset R125 using existing methods. Number of total singlets and the singlet rate were highlighted in bold.

|  | **Multi-seq** | **GMM_demux** | **BFF_raw** | **BFF_cluster** | **DropletUtils** | **HTOreader** | **HTOdemux (Seurat)** |
| --- | --- | --- | --- | --- | --- | --- | --- |
| Doublet | 70 | 63 | 52 | 47 | 66 | 78 | 66 |
| Hashtag1 | 166 | 156 | 153 | 171 | 160 | 160 | 169 |
| Hashtag2 | 97 | 445 | 416 | 523 | 432 | 437 | 218 |
| Hashtag3 | 229 | 216 | 218 | 241 | 224 | 215 | 229 |
| Negative | 427 | 109 | 150 | 7 | 107 | 99 | 307 |
| **singlet** | **492** | **817** | **787** | **935** | **816** | **812** | **616** |
| **singlet rate** | **49.75%** | **82.61%** | **79.58%** | **94.54%** | **82.51%** | **82.10%** | **62.29%** |

**Table S8.** Performance of tested methods of dataset R125 when validating with ground truth.

|  | **Acc** | **Recall** | **Precision** | **F1** | **singlet rate** |
| --- | --- | --- | --- | --- | --- |
| **Multi-seq** | 0.48593074 | 0.48468708 | 0.80530973 | 0.60515378 | 0.48917749 |
| **GMM_demux** | 0.66450216 | 0.81121751 | 0.7751634 | 0.79278075 | 0.82792208 |
| **BFF_raw** | 0.64502165 | 0.78219178 | 0.77162162 | 0.77687075 | 0.8008658 |
| **BFF_cluster** | 0.75649351 | 0.94751381 | 0.78579611 | 0.85911083 | 0.94480519 |
| **Dropletutils** | 0.65909091 | 0.80547945 | 0.77266754 | 0.78873239 | 0.82359307 |
| **HTOreader** | 0.66233766 | 0.80684932 | 0.775 | 0.79060403 | 0.82251082 |
| **HTOdemux(Seurat)** | 0.55194805 | 0.60845839 | 0.77835951 | 0.68300153 | 0.62012987 |

**Table S9**. Correlation between Souporcell genotype clusters and BFF_raw method of dataset 8pool-CA.

|  | doublet | singlet 0 | singlet 1 | singlet 2 | singlet 3 | singlet 4 | singlet 5 | singlet 6 | singlet 7 | unassigned |
| --- | --- | --- | --- | --- | --- | --- | --- | --- | --- | --- |
| Doublet | 1090 | 145 | 68 | 80 | 93 | 61 | 73 | 26 | 63 | 5 |
| Negative | 59 | 45 | 8 | 55 | 11 | 37 | 37 | 227 | 58 | 1 |
| S282 | 0 | 0 | 0 | 0 | 0 | 0 | 0 | 0 | 0 | 0 |
| S283 | 33 | 0 | 1 | 1 | 1755 | 0 | 0 | 4 | 0 | 0 |
| S284 | 28 | 0 | 0 | 1494 | 0 | 1 | 0 | 1 | 1 | 0 |
| S344 | 33 | 0 | 1537 | 0 | 1 | 2 | 0 | 10 | 0 | 0 |
| S397 | 18 | 2053 | 0 | 0 | 0 | 0 | 0 | 7 | 2 | 0 |
| S417 | 30 | 0 | 0 | 0 | 1 | 1155 | 1 | 7 | 0 | 2 |
| S421 | 5 | 0 | 0 | 0 | 0 | 0 | 0 | 2 | 784 | 0 |
| S423 | 30 | 0 | 0 | 0 | 0 | 0 | 1639 | 6 | 0 | 0 |

**Table S10**. Correlation between Souporcell genotype clusters and BFF_cluster method of dataset 8pool-CA.

|  | doublet | singlet 0 | singlet 1 | singlet 2 | singlet 3 | singlet 4 | singlet 5 | singlet 6 | singlet 7 | unassigned |
| --- | --- | --- | --- | --- | --- | --- | --- | --- | --- | --- |
| Doublet | 475 | 28 | 15 | 26 | 34 | 16 | 26 | 14 | 17 | 2 |
| Negative | 59 | 45 | 6 | 55 | 11 | 31 | 29 | 227 | 54 | 1 |
| S282 | 0 | 0 | 0 | 0 | 0 | 0 | 0 | 0 | 0 | 0 |
| S283 | 139 | 2 | 5 | 2 | 1804 | 3 | 0 | 4 | 1 | 1 |
| S284 | 118 | 5 | 2 | 1536 | 2 | 1 | 0 | 1 | 3 | 2 |
| S344 | 121 | 2 | 1579 | 1 | 4 | 4 | 3 | 15 | 0 | 0 |
| S397 | 135 | 2152 | 3 | 3 | 1 | 2 | 3 | 10 | 4 | 0 |
| S417 | 114 | 2 | 1 | 1 | 3 | 1194 | 2 | 9 | 3 | 2 |
| S421 | 41 | 0 | 1 | 4 | 1 | 2 | 4 | 2 | 824 | 0 |
| S423 | 124 | 7 | 2 | 2 | 1 | 3 | 1683 | 8 | 2 | 0 |

**Table S11**. Correlation between Souporcell genotype clusters and HTOdemux(Seurat) method of dataset 8pool-CA.

|  | doublet | singlet 0 | singlet 1 | singlet 2 | singlet 3 | singlet 4 | singlet 5 | singlet 6 | singlet 7 | unassigned |
| --- | --- | --- | --- | --- | --- | --- | --- | --- | --- | --- |
| Doublet | 1213 | 588 | 163 | 167 | 176 | 123 | 125 | 58 | 141 | 5 |
| Negative | 4 | 27 | 1 | 76 | 1 | 5 | 12 | 1 | 0 | 0 |
| S282 | 37 | 0 | 0 | 1 | 0 | 0 | 0 | 220 | 43 | 1 |
| S283 | 14 | 0 | 0 | 1 | 1682 | 0 | 0 | 1 | 0 | 0 |
| S284 | 4 | 0 | 0 | 1385 | 0 | 0 | 0 | 0 | 0 | 0 |
| S344 | 18 | 0 | 1450 | 0 | 1 | 0 | 0 | 7 | 0 | 0 |
| S397 | 7 | 1627 | 0 | 0 | 0 | 0 | 0 | 1 | 0 | 0 |
| S417 | 13 | 1 | 0 | 0 | 1 | 1128 | 1 | 2 | 0 | 2 |
| S421 | 3 | 0 | 0 | 0 | 0 | 0 | 0 | 0 | 724 | 0 |
| S423 | 13 | 0 | 0 | 0 | 0 | 0 | 1612 | 0 | 0 | 0 |

**Table S12**. Correlation between Souporcell genotype clusters and MULTI_seq method of dataset 8pool-CA.

|  | doublet | singlet 0 | singlet 1 | singlet 2 | singlet 3 | singlet 4 | singlet 5 | singlet 6 | singlet 7 | unassigned |
| --- | --- | --- | --- | --- | --- | --- | --- | --- | --- | --- |
| Doublet | 1182 | 335 | 92 | 92 | 120 | 76 | 82 | 51 | 85 | 5 |
| Negative | 4 | 61 | 5 | 3 | 21 | 5 | 43 | 16 | 1 | 0 |
| S282 | 48 | 0 | 0 | 0 | 0 | 0 | 1 | 211 | 47 | 1 |
| S283 | 20 | 0 | 1 | 0 | 1718 | 0 | 0 | 1 | 0 | 0 |
| S284 | 9 | 0 | 0 | 1535 | 0 | 0 | 0 | 1 | 0 | 0 |
| S344 | 22 | 0 | 1516 | 0 | 1 | 0 | 1 | 8 | 0 | 0 |
| S397 | 8 | 1846 | 0 | 0 | 0 | 0 | 1 | 0 | 0 | 0 |
| S417 | 15 | 1 | 0 | 0 | 1 | 1175 | 1 | 2 | 0 | 2 |
| S421 | 4 | 0 | 0 | 0 | 0 | 0 | 0 | 0 | 775 | 0 |
| S423 | 14 | 0 | 0 | 0 | 0 | 0 | 1621 | 0 | 0 | 0 |

**Table S13**. Correlation between Souporcell genotype clusters and GMM_Demux method of dataset 8pool-CA.

|  | doublet | singlet 0 | singlet 1 | singlet 2 | singlet 3 | singlet 4 | singlet 5 | singlet 6 | singlet 7 | unassigned |
| --- | --- | --- | --- | --- | --- | --- | --- | --- | --- | --- |
| Doublet | 1215 | 532 | 155 | 131 | 172 | 103 | 117 | 59 | 134 | 5 |
| Negative | 4 | 9 | 0 | 5 | 2 | 1 | 6 | 0 | 0 | 0 |
| S282 | 44 | 0 | 0 | 0 | 0 | 0 | 0 | 220 | 45 | 1 |
| S283 | 11 | 0 | 1 | 0 | 1685 | 0 | 0 | 1 | 0 | 0 |
| S284 | 5 | 0 | 0 | 1494 | 0 | 0 | 0 | 1 | 0 | 0 |
| S344 | 18 | 0 | 1457 | 0 | 1 | 0 | 0 | 8 | 0 | 0 |
| S397 | 6 | 1702 | 0 | 0 | 0 | 0 | 0 | 0 | 0 | 0 |
| S417 | 10 | 0 | 0 | 0 | 1 | 1151 | 1 | 1 | 0 | 2 |
| S421 | 1 | 0 | 0 | 0 | 0 | 0 | 0 | 0 | 729 | 0 |
| S423 | 12 | 0 | 0 | 0 | 0 | 0 | 1626 | 0 | 0 | 0 |

**Table S14**. Hybrid demultiplexing on all cells of dataset 9pool-CA. The convergence score between cell hashing-based and SNP-based demultiplexing is 0.1.

|  | | Hashtag demultiplexing | | | | | | | | | | |
| --- | --- | --- | --- | --- | --- | --- | --- | --- | --- | --- | --- | --- |
|  |  | Doublet | S289 | S297 | S299 | S302 | S354 | S365 | S374 | S403 | S409 | Negative |
| SNP demultiplexing | doublet | 488 | 161 | 102 | 96 | 58 | 104 | 147 | 142 | 222 | 169 | 38 |
|  | singlet0 | 134 | 39 | 27 | 34 | 26 | 27 | 42 | 35 | 63 | 58 | 7 |
|  | singlet1 | 624 | 195 | 139 | 137 | 78 | 148 | 171 | 200 | 321 | 237 | 60 |
|  | singlet2 | 371 | 178 | 62 | 83 | 44 | 60 | 106 | 123 | 164 | 142 | 19 |
|  | singlet3 | 261 | 81 | 86 | 72 | 28 | 64 | 75 | 80 | 146 | 93 | 12 |
|  | singlet4 | 462 | 129 | 108 | 103 | 60 | 87 | 120 | 146 | 195 | 225 | 34 |
|  | singlet5 | 237 | 83 | 55 | 68 | 29 | 61 | 76 | 63 | 114 | 102 | 13 |
|  | singlet6 | 372 | 106 | 91 | 82 | 41 | 82 | 90 | 152 | 178 | 126 | 20 |
|  | singlet7 | 293 | 98 | 73 | 68 | 34 | 63 | 115 | 94 | 141 | 128 | 22 |
|  | singlet8 | 570 | 216 | 141 | 105 | 53 | 105 | 138 | 158 | 355 | 224 | 41 |
|  | unassigned | 98 | 25 | 29 | 16 | 15 | 17 | 23 | 27 | 47 | 34 | 11 |

**Table S15**. Hybrid demultiplexing on dataset 9pool-CA after remove all B&T doublets. The convergence score between cell hashing-based and SNP-based demultiplexing is 0.55.

|  | | Hashtag demultiplexing | | | | | | | | | | |
| --- | --- | --- | --- | --- | --- | --- | --- | --- | --- | --- | --- | --- |
|  |  | Doublet | S289 | S297 | S299 | S302 | S354 | S365 | S374 | S403 | S409 | Negative |
| SNP demultiplexing | doublet | 344 | 50 | 48 | 39 | 18 | 56 | 50 | 47 | 70 | 49 | 6 |
|  | singlet0 | 214 | 116 | 55 | 54 | 50 | 41 | 60 | 72 | 96 | 930 | 10 |
|  | singlet1 | 185 | 521 | 99 | 74 | 45 | 48 | 80 | 105 | 131 | 88 | 14 |
|  | singlet2 | 94 | 74 | 25 | 34 | 145 | 22 | 35 | 42 | 43 | 50 | 4 |
|  | singlet3 | 137 | 83 | 388 | 45 | 32 | 30 | 67 | 81 | 60 | 57 | 4 |
|  | singlet4 | 80 | 44 | 23 | 31 | 16 | 381 | 38 | 32 | 37 | 20 | 6 |
|  | singlet5 | 123 | 51 | 39 | 406 | 32 | 36 | 69 | 57 | 47 | 49 | 1 |
|  | singlet6 | 143 | 67 | 63 | 49 | 35 | 52 | 510 | 85 | 66 | 71 | 9 |
|  | singlet7 | 258 | 127 | 69 | 57 | 50 | 57 | 62 | 85 | 1262 | 94 | 15 |
|  | singlet8 | 168 | 94 | 61 | 63 | 31 | 57 | 90 | 573 | 89 | 77 | 11 |
|  | unassigned | 92 | 24 | 25 | 3 | 8 | 31 | 23 | 32 | 30 | 39 | 5 |

**Table S16**. Hybrid demultiplexing on dataset 9pool-CA after remove all doublets identified by cell hashing method. The convergence score between cell hashing-based and SNP-based demultiplexing is 0.95.

|  | | Hashtag demultiplexing | | | | | | | | | | |
| --- | --- | --- | --- | --- | --- | --- | --- | --- | --- | --- | --- | --- |
|  |  | Doublet | S289 | S297 | S299 | S302 | S354 | S365 | S374 | S403 | S409 | Negative |
| SNP demultiplexing | doublet | 0 | 15 | 12 | 17 | 8 | 13 | 17 | 13 | 28 | 14 | 26 |
|  | singlet0 | 0 | 0 | 0 | 32 | 414 | 0 | 0 | 0 | 0 | 0 | 4 |
|  | singlet1 | 0 | 0 | 0 | 0 | 0 | 47 | 999 | 0 | 0 | 1 | 6 |
|  | singlet2 | 0 | 0 | 0 | 0 | 0 | 0 | 0 | 1 | 91 | 1382 | 26 |
|  | singlet3 | 0 | 0 | 23 | 809 | 0 | 0 | 0 | 2 | 0 | 0 | 7 |
|  | singlet4 | 0 | 0 | 0 | 0 | 0 | 1 | 59 | 1100 | 0 | 0 | 6 |
|  | singlet5 | 0 | 14 | 852 | 0 | 0 | 0 | 0 | 0 | 0 | 0 | 5 |
|  | singlet6 | 0 | 0 | 0 | 0 | 37 | 730 | 0 | 0 | 1 | 0 | 10 |
|  | singlet7 | 0 | 1205 | 0 | 0 | 0 | 0 | 0 | 0 | 0 | 0 | 9 |
|  | singlet8 | 0 | 0 | 0 | 0 | 0 | 0 | 1 | 71 | 1798 | 0 | 30 |
|  | unassigned | 0 | 77 | 26 | 6 | 7 | 27 | 27 | 33 | 28 | 141 | 148 |
